# Whole-body deletion of Endospanin 1 protects from obesity-associated deleterious metabolic alterations

**DOI:** 10.1172/jci.insight.168418

**Published:** 2024-05-08

**Authors:** Arturo Roca-Rivada, Marcio Do Cruzeiro, Raphaël G.P. Denis, Qiang Zhang, Christine Rouault, Yves Rouillé, Jean-Marie Launay, Céline Cruciani-Guglielmacci, Virginie Mattot, Karine Clément, Ralf Jockers, Julie Dam

**Affiliations:** 1 Institut Cochin, Inserm U1016, CNRS UMR 8104, Université Paris Cité, F-75014 Paris, France.; 2 Unité de Biologie Fonctionnelle et Adaptative, Université Paris Cité, CNRS, 75013 Paris, France.; 3 Sorbonne Université, Inserm, Nutrition and obesities: systemic approaches, Nutriomics, Department of Nutrition, Pitié-Salpêtrière Hospital, Assistance Publique Hopitaux de Paris, Paris, France.; 4 Université de Lille, CNRS, INSERM, CHU Lille, Institut Pasteur de Lille, U1019 - UMR 9017 - CIIL - Center for Infection and Immunity of Lille, F-59000, Lille, France.; 5 Université Paris Cité, Inserm UMR-S 942, Paris, France.; 6 Université Paris Cité, Inserm, CHU Lille, Laboratory of Development and Plasticity of the Neuroendocrine Brain, Lille Neuroscience & Cognition, UMR-S1172, EGID, F-59000, Lille, France.

**Keywords:** Cell biology, Metabolism, Adipose tissue, Diabetes, Obesity

## Abstract

The importance of the proper localization of most receptors at the cell surface is often underestimated, although this feature is essential for optimal receptor response. Endospanin 1 (Endo1) (also known as OBRGRP or LEPROT) is a protein generated from the same gene as the human leptin receptor and regulates the trafficking of proteins to the surface, including the leptin receptor. The systemic role of Endo1 on whole-body metabolism has not been studied so far. Here, we report that general Endo1-KO mice fed a high-fat diet develop metabolically healthy obesity with lipid repartitioning in organs and preferential accumulation of fat in adipose tissue, limited systematic inflammation, and better controlled glucose homeostasis. Mechanistically, Endo1 interacts with the lipid translocase CD36, thus regulating its surface abundance and lipid uptake in adipocytes. In humans, the level of Endo1 transcripts is increased in the adipose tissue of patients with obesity, but low levels rather correlate with a profile of metabolically healthy obesity. We suggest here that Endo1, most likely by controlling CD36 cell surface abundance and lipid uptake in adipocytes, dissociates obesity from diabetes and that its absence participates in metabolically healthy obesity.

## Abstract 2

Endospanin 1 controls surface abundance of lipid translocase CD36 in adipocytes, which allows dissociation of obesity from diabetes, and its reduction is linked to metabolically healthy obesity.

## Graphical abstract

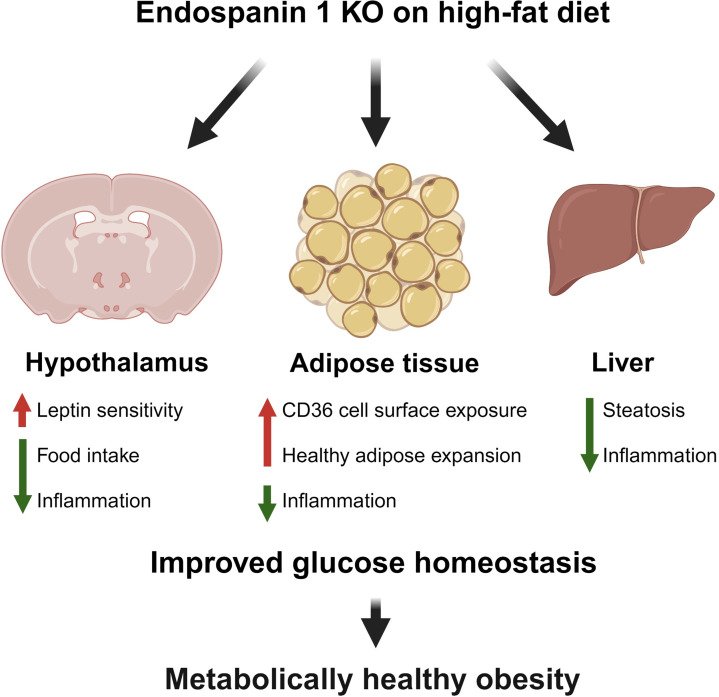

## Introduction

Obesity is a metabolic condition in which excessive lipid accumulation in the adipose tissue and various organs leads to the development of a multitude of metabolic diseases, including impaired glucose tolerance, type 2 diabetes (T2D), dyslipidemia, hypertension, and liver and cardiovascular diseases (1). Obesity is characterized by exacerbated adipose tissue expansion and dysfunction, chronic inflammation, and fibrosis. However, a healthier expansion of the adipose tissue in the context of obesity has also been reported, suggesting the possibility of dissociating obesity from metabolic disorders (2). Metabolically healthy obesity (MHO) has been recognized (3), but the mechanisms underlying MHO with preserved metabolic functions are still unclear.

The adipose tissue produces and releases into the circulation a multitude of signals, including the adipokine leptin (4), that can regulate whole-body energy homeostasis. Activation of the leptin receptor (LEPR), a type I cytokine receptor, in neurons of the arcuate nucleus of the hypothalamus (ARH) suppresses food intake, induces energy expenditure, and promotes glycemic control (5, 6). Despite high circulating leptin levels, this hormone is unable to function properly during obesity, leading to a state of leptin resistance. One of the underlying mechanisms behind leptin resistance is a defective trafficking of the leptin receptor to the cell surface, affecting its proper signaling (7, 8). Of interest, the human *LEPR* gene can generate, by alternative splicing, a series of overlapping *LEPR* transcripts coding for different leptin receptor isoforms and the leptin receptor overlapping transcript (*LEPROT*), a transcript encoding a 4-transmembrane protein called endospanin 1 (Endo1), displaying no sequence similarity to the leptin receptor itself (9, 10). In contrast to humans, in mice, *Lepr* and *Leprot* transcripts are generated from 2 independent genes. Endo1, also known as leptin receptor gene-related protein (OB-RGRP), is well conserved from yeast to mammals (11). The identification of a yeast homolog of Endo1 — Vps55p, a protein involved in the regulation of membrane trafficking between endosomes and vacuoles — suggested that Endo1 may also be involved in intracellular protein trafficking (12). In agreement with this hypothesis, Endo1 was mainly located in the trans-Golgi network and endosomes (13). Accordingly, Endo1 has been shown to regulate the targeting of the growth hormone receptor to the plasma membrane and its functional response in the liver (14) as well as the cell surface pool of the leptin receptor and its function in the hypothalamus (15–17). More recently, Endo1 has been identified as a regulator of receptor-mediated transcytosis in polarized cells, and its absence can affect the trafficking mechanism in these cells (18).

Interestingly, silencing of Endo1 in the ARH has a dual effect. It prevents diet-induced obesity (DIO) by increasing leptin signaling through the STAT3 pathway (15, 16), but it participates in glucose intolerance (GI) by decreasing leptin signaling through the PI3K/AKT pathway (17). The effect on the STAT3 pathway results from the downregulation of LEPR trafficking to the cell surface by Endo1 (15, 16), while the effect on the PI3K pathway results from an interaction of Endo1 with the regulatory subunit of PI3K (17). However, Endo1 expression is not only restricted to the ARH; we also observed its expression in other organs, including the adipose tissue. Little is known about the metabolic importance of Endo1 and its integrated function in the whole organism.

In the present study, we analyze the effects of an Endo1-KO mouse model (referred to as Endo KO) with a global deletion of Endo1. The absence of this protein in the whole body enhances several processes that can be related to increased leptin receptor function such as decreased food intake and expression of orexigenic neuropeptides. In addition, deletion of Endo1 increases the cell surface localization of the lipid transporter CD36 in the adipose tissue, thereby inducing an increase in its lipid uptake capacity, reducing ectopic fat accumulation in the liver, and limiting HFD-related inflammation. Importantly, the conjunction of these events improves HFD-related glucose homeostasis in Endo1 KO. These observations in obese Endo1-KO animals are also supported by data in obese humans. Overall, we show that the global deletion of Endo1 is more likely to promote MHO.

## Results

### Tissue expression of Endo1.

To understand the function of Endo1 in metabolic regulation, we generated mice deleted for its *Leprot* gene (Endo1 KO). *Lepr* mRNA levels were not modified in the hypothalamus of Endo1-KO mice (Supplemental Figure 1A; supplemental material available online with this article; https://doi.org/10.1172/jci.insight.168418DS1). Ablation of Endo1 expression in Endo1-KO animals was confirmed at the mRNA and protein levels in multiple tissues (including hypothalamus, adipose tissues, and liver) (Supplemental Figure 1, B and C). Similar levels of fasted metabolic hormones (leptin, insulin, C-peptide, glucagon, ghrelin, GLP1, amylin) in Endo1-KO or WT mice suggests that the absence of Endo1 does not affect basal levels of metabolic hormones in the plasma (Supplemental Figure 2A). Since we previously observed an effect of Endo1 on the anorexigenic leptin receptor (15–17), we assessed food intake in Endo1-KO mice. A decrease in the orexigenic AGRP and a trend toward a decrease in NPY transcripts in the ARH of Endo1-KO mice was detected after 12-hour fasting (Supplemental Figure 2B). The levels of orexigenic neuropeptide transcripts correlated with reduced food intake of Endo1 KO (Supplemental Figure 2C), suggesting an improved endogenous leptin signaling in Endo1-KO mice. In line with this, exogenously administered leptin decreased food intake in Endo1-KO versus WT mice (Supplemental Figure 2D), as previously observed with the ARH-Endo1 silencing (16, 17). Altogether, the data suggest that the Endo1-KO mouse model recapitulates the reduced food intake observed after Endo1 silencing in the hypothalamic ARH.

In the mouse hypothalamus, we detected Endo1 protein expression in the ARH and in the ventromedial nucleus of the hypothalamus (VMH) and to a much lesser extent in the dorsomedial nucleus (DMH) (Supplemental Figure 3). However, in a broader tissue expression profiling of Endo1, the hypothalamus was not the predominant expression tissue. Indeed, we observed that the abundance of the *Leprot* transcript (Supplemental Figure 1B), and of Endo1 protein (Supplemental Figure 1, C and D), differed in various metabolic tissues, with highest levels in the adipose tissues (s.c. adipose tissue [SAT], gonadal adipose tissue [GAT]) and lungs followed by hypothalamus, liver, and pancreas, with the lowest expression in muscle (Supplemental Figure 1D).

### Endo1 interacts with CD36 in the adipose tissue and controls its localization at the surface of adipocytes.

**Figure 1:**
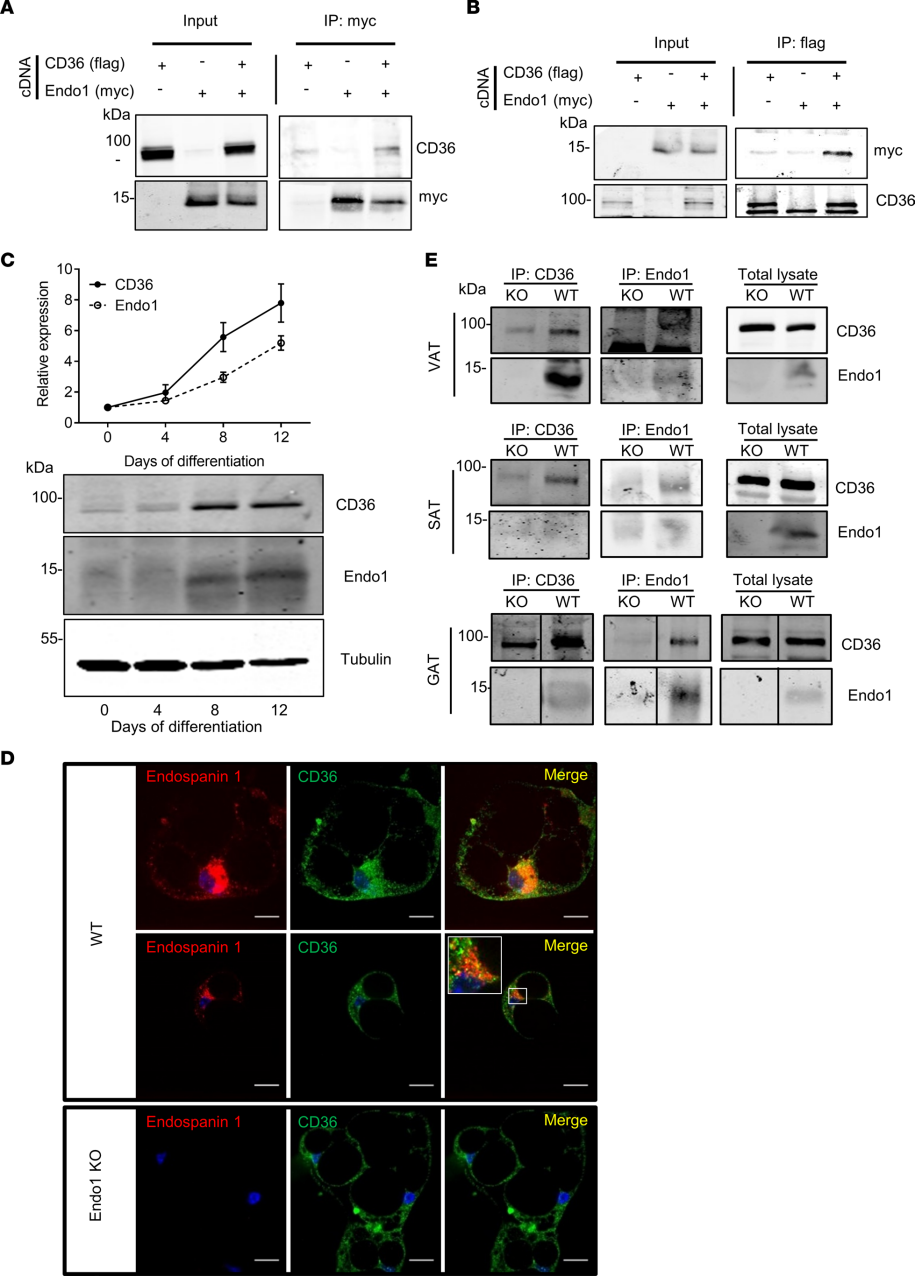
Endospanin 1 directly interacts with CD36. (**A**) Detection of Flag-CD36 in 6Myc-Endo1 immunoprecipitate from HEK293T cells coexpressing 6Myc-Endo1 and Flag-CD36 compared with HEK293T cells transfected with Flag-CD36 alone. Representative blots of 3 independent experiments. (**B**) Detection of 6Myc-Endo1 after Flag-CD36 immunoprecipitation from HEK293T cells coexpressing 6Myc-Endo1 and Flag-CD36 compared with HEK293T cells transfected with 6Myc-Endo1 alone. Representative blots of 3 independent experiments. (**C**) Kinetics of Endo1 and CD36 protein expression in differentiated adipocytes during differentiation of adipocyte precursors isolated from the stromal vascular fraction of the s.c. adipose tissue of WT mice into mature white adipocytes. Results are expressed as mean ± SEM of 8 independent experiments. One representative Western blot is shown. (**D**) Confocal immunofluorescence detection and colocalization of Endo1 (rabbit anti-Endo1) and CD36 (goat anti-CD36) in differentiated adipocytes. Nuclei (blue) are stained with fluorescent DAPI dye. Scale bar: 20 μm. Representative images of 4 independent experiments. (**E**) Detection of endogenous Endo1 and CD36 after immunoprecipitation with Endo1 or CD36 antibodies from lysates of visceral adipose tissue (VAT), gonadal adipose tissue (GAT), and s.c. adipose tissue (SAT) of WT and KO mice. Endo1-KO adipocytes and tissues were used as negative controls. The molecular weights of protein markers are indicated (kDa). Representative blots of 2 independent experiments.

Among the different adipose tissues, Endo1 transcript and protein are found to be more expressed in the SAT, known to provide beneficial metabolic parameters, and are less expressed in visceral adipose tissue (VAT), known to exert deleterious metabolic functions in obesity (Supplemental Figure 1, B–D). Expression of Endo1 in adipose tissues is interesting, considering that our coimmunoprecipitation (co-IP) experiments between Endo1 and several receptors suggested a possible interaction between Endo1 and CD36, the first mammalian plasma membrane fatty acid transporter discovered and characterized in adipose tissue (19) (Figure 1, A and B). In our co-IP experiments, Flag-tagged CD36 was immunoprecipitated along with 6myc-tagged Endo1 expressed in HEK293T cells while it was absent in the immunoprecipitate when 6myc-Endo1 was not expressed (Figure 1A). Conversely, 6myc-Endo1 was readily immunoprecipitated with Flag-CD36 when both were expressed in HEK293T cells, whereas 6myc-Endo1 was not immunoprecipitated when Flag-CD36 was absent (Figure 1B). This suggests that 6myc-Endo1 and Flag-CD36 form a stable molecular complex in transfected HEK293T cells. We became intrigued by this result, as an examination of the public database (20) reveals that *LEPROT* and *CD36* are 2 genes primarily expressed in human adipose tissue compared with other organs (Supplemental Figure 4A). Single-cell RNA-Seq analysis suggested that the highest expression of these 2 genes was predominantly found in adipocytes and fibroblasts within the human adipose tissue (Supplemental Figure 4B). This observation was further corroborated by the analysis of a recently published database (21), which shows a similar expression pattern between *CD36* and *LEPROT* in single-cell analysis of human adipose tissue, where adipocytes or adipose stem precursor cells (ASPC) rank among the cell types in which *CD36* and *LEPROT* are highly expressed (Supplemental Figure 4C). Notably, we also detected a subset of human adipocytes in which the transcript levels of *CD36* and *LEPROT* were positively correlated (Supplemental Figure 4D).

Since, at the protein level in mice, CD36 and Endo1 were mainly expressed in the adipose tissue and, to a lower extent, in the hypothalamus, liver, and muscle (Supplemental Figure 1D), we concentrated our effort on adipocytes. We confirmed that CD36 and Endo1 proteins were both endogenously expressed in mouse adipocytes with increasing protein expression observed during the differentiation of adipocyte precursors from the stromal vascular fraction of SAT into white adipocytes (Figure 1C). The main fraction of Endo1 in adipocytes was detected in the Golgi (Supplemental Figure 5A), similarly to what was previously observed in HeLa cells (13). The differentiation process was not affected in adipocyte precursors of Endo1-KO mice, suggesting that Endo1 was not crucial for the differentiation process (Supplemental Figure 5, B and C). Expression of CD36 and Endo1 in differentiated white adipocytes was further confirmed by confocal microscopy, revealing that both proteins colocalized in intracellular compartments (Figure 1D). CD36 interacted endogenously with Endo1 in GAT, VAT, and SAT, as shown by immunoprecipitation of endogenous CD36 or Endo1 in adipose tissues of WT mice but not Endo1-KO mice (Figure 1E). This interaction appears to have been specific to adipose tissues, as we failed to detect it in the liver (Supplemental Figure 6).

**Figure 2:**
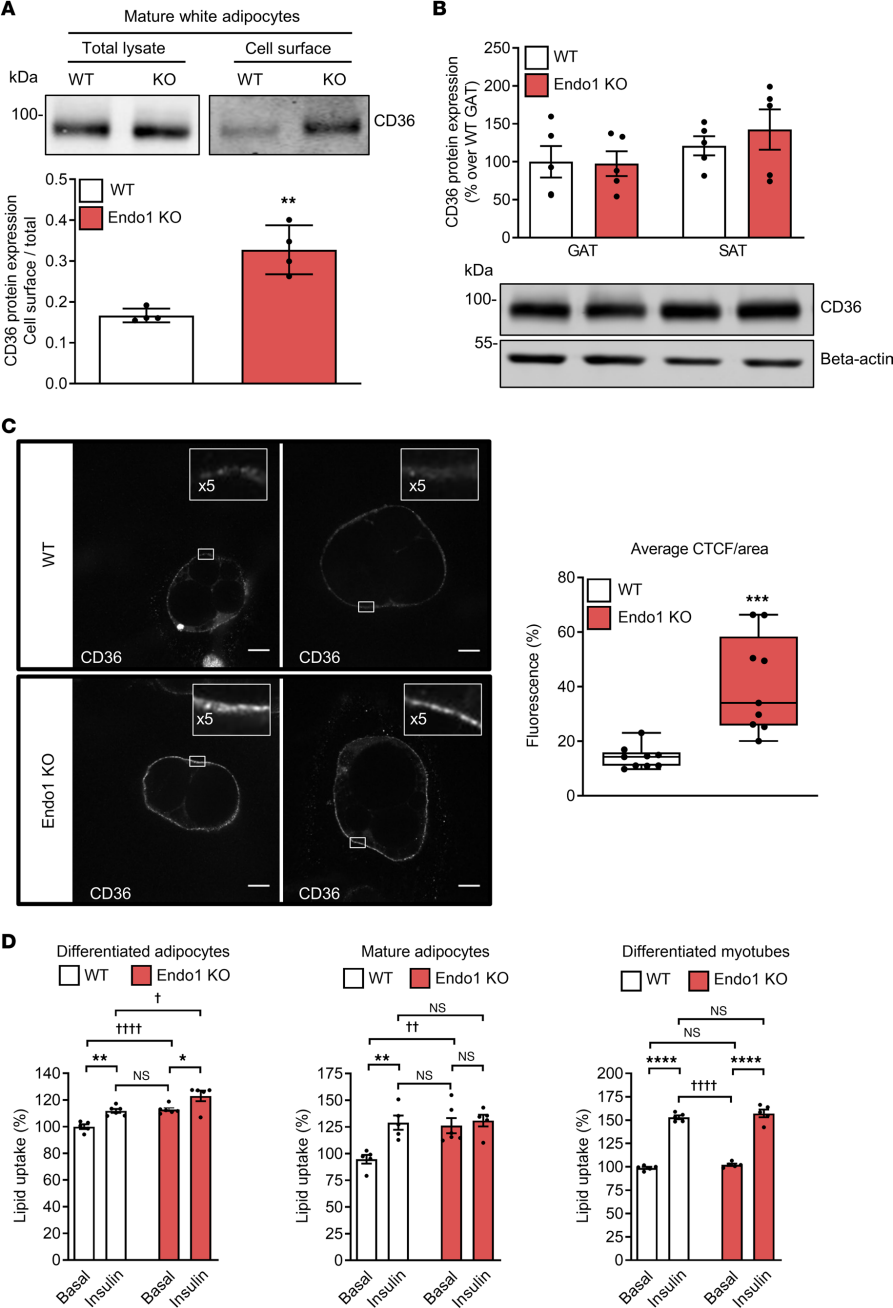
Absence of Endo1 increases cell surface CD36 levels and lipid uptake in adipocytes. (**A**) Cell surface expression of CD36 in mature white adipocytes. **P < 0.01 versus WT. Results are expressed as mean ± SEM (*n* = 4). Two-tailed *t* test. (**B**) Total CD36 expression in gonadal adipose tissue (GAT) and s.c. adipose tissue (SAT) . The molecular weights of protein markers are indicated (kDa). Results are expressed as mean ± SEM (*n* = 5). (**C**) Immunofluorescence images of CD36 cell surface expression in differentiated white adipocytes (left panel). Level of cellular fluorescence determined by corrected total cell fluorescence per area (CTCF/area). Results are expressed as mean ± SEM (*n* = 9). ***P < 0.005 versus WT. Two-tailed *t* test (right panel). Scale bar: 20 μm. (**D**) Lipid uptake in differentiated adipocytes, mature adipocytes, and differentiated myotubes. Results are expressed as mean ± SEM (*n* = 5–6). *P < 0.05; **P < 0.01; ****P < 0.001 versus basal. ^†^*P* < 0.05; ^††^*P* < 0.01; ^††††^*P* < 0.001 versus WT. One-way ANOVA with Bonferroni correction.

We then asked the question of whether Endo1 regulates the fraction of CD36 available at the surface of adipocytes. Biotinylation of adipocyte surface proteins shows that the level of CD36 at the plasma membrane was increased in mature white adipocytes from Endo1-KO SAT compared with WT adipocytes (Figure 2A), while the total protein level of CD36 was similar in mature adipocytes (Figure 2A) and in the adipose tissues of both mouse strains (Figure 2B). Consistently, CD36 labeling on the surface of nonpermeabilized adipocytes from Endo1-KO mice was twice as intense as that of WT adipocytes (Figure 2C). CD36 is a lipid transporter, the level of which on the cell surface will determine the level of lipid uptake by these cells. Consistent with an increased surface localization of CD36, mature adipocytes derived from adipose tissue of Endo1-KO mice, or Endo1-KO precursors differentiated into white adipocytes, take up more lipids in the basal state and after stimulation with insulin (Figure 2D). No difference in lipid uptake was observed in Endo1-KO and WT muscle cells differentiated into myotubes (Figure 2D), consistent with the observation of approximately 5–6 times lower Endo1 expression levels in the gastrocnemius muscle compared with SAT (Supplemental Figure 1D).

Taken together, these data indicate that Endo1 regulates the number of cell surface CD36 by retaining CD36 in intracellularly compartments similar to previous observations made with the leptin receptor (13). This effect is particularly pronounced in adipocytes expressing high CD36 and Endo1 levels. Decreasing levels of Endo1 in adipocytes increases the surface expression of CD36 and amplifies lipid uptake by adipocytes. We hypothesize that this particular process would, therefore, favor lipid uptake into the adipose tissue of mice challenged by a lipid-rich diet.

### Endo1 deletion in Endo1-KO mice promotes increased adipose tissue in obesity.

**Figure 3:**
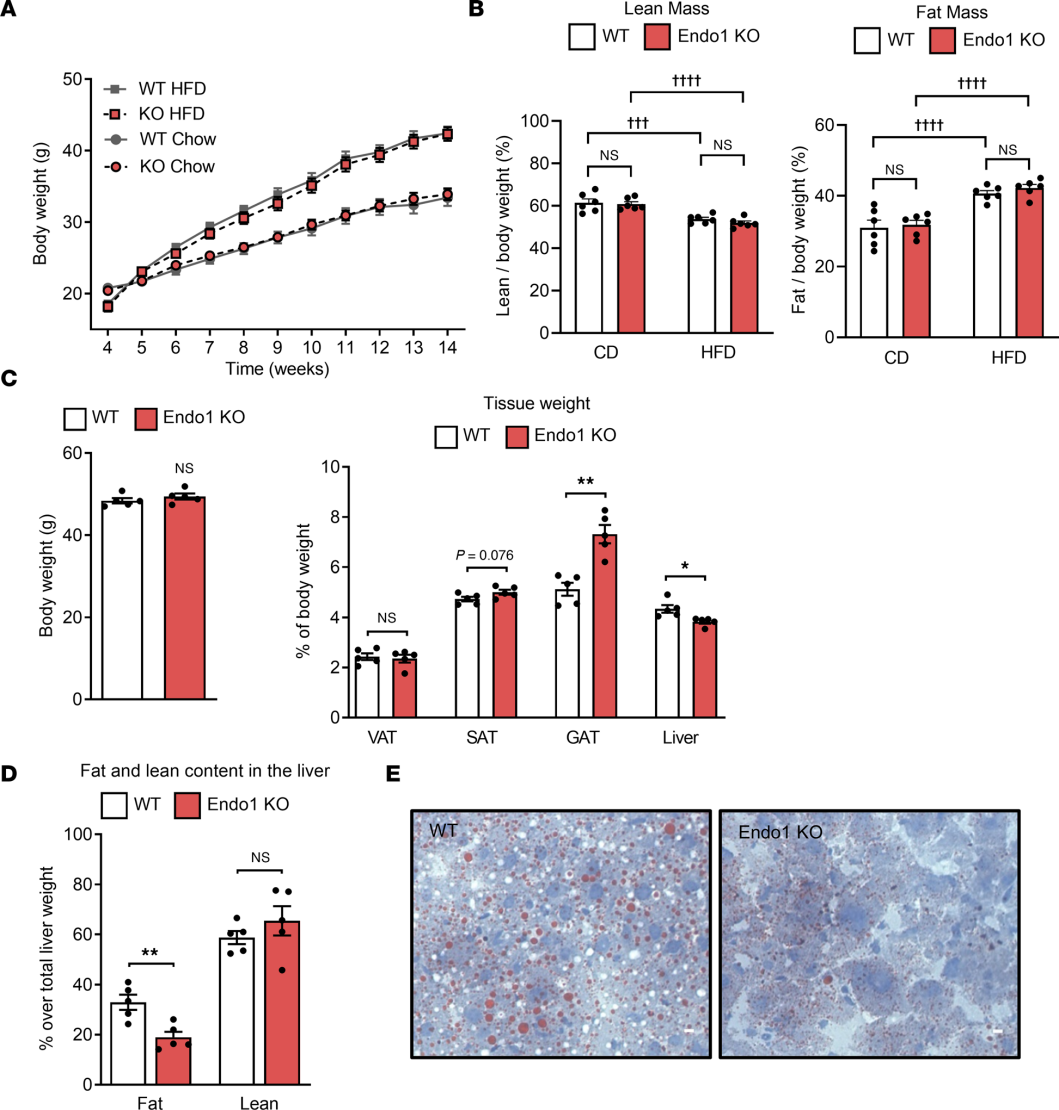
Body weight and fat distribution in Endo1-KO and WT mice fed a standard chow diet (CD) or a high-fat diet (HFD). (**A**) Body weight monitored for 10 weeks. Results are expressed as mean ± SEM (*n* = 10–12). (**B**) Total fat and lean mass content normalized to total body weight (%) in CD and HFD. Results are expressed as mean ± SEM (*n* = 6). ^†††^*P* < 0.001; ^††††^*P* < 0.0001 versus CD. One-way ANOVA with Tukey’s test. (**C**) Adipose tissue weight distribution. Results are expressed as mean ± SEM (*n* = 5). *P < 0.05; **P < 0.01 versus WT. Two-tailed *t* test. (**D**) Fat and lean content in liver after 4 weeks of HFD measured by NMR. Results are expressed as mean ± SEM (*n* = 5). **P < 0.01 versus WT. Two-tailed *t* test. (**E**) Oil Red O staining of liver sections after 4 weeks of HFD. Representative images of 5 mice of each genotype. Scale bar: 50 μm.

To explore the hypothesis that deletion of Endo1 may promote lipid uptake in the adipose tissue, Endo1-KO mice were subjected to a high-fat diet–induced (HFD-induced) obesity model and to a chow diet (CD). In agreement with our previous data on increased leptin sensitivity, during HFD, under Endo1 knockdown in the ARH at the level of STAT3 pathway (16), we confirmed a higher phosphorylation of endogenous STAT3 in the hypothalamus of Endo1-KO mice under HFD compared with WT controls (Supplemental Figure 7A). Accordingly, Endo1-KO mice ate less than WT mice (Supplemental Figure 7, B–D). Calorimetric investigations of the metabolic parameters of both strains in metabolic cages showed similar energy expenditure (Supplemental Figure 7, E–G), locomotor activity (Supplemental Figure 7H), and respiratory exchange ratio (RER) (Supplemental Figure 7I) in both diets, with an energy balance tending to be less positive than WT on HFD but not significantly (Supplemental Figure 7J). The body weight and fat mass of the 2 strains were, therefore, similar in the 2 diets (Figure 3A), correlating with similar fasting leptinemia known to be related to fat levels (Supplemental Figure 8A), with no significant variation in lean mass or overall fat mass (Figure 3B and Supplemental Figure 8B). However, upon detailed analysis of fat partitioning in obese animals, Endo1-KO mice show a redistribution of fat, with increased s.c. fat (trend, *P* = 0.076), increased gonadal depot (*P* = 0.001), and a significant decrease in liver fat (*P* = 0.016) (Figure 3, C–E, and Supplemental Figure 8, C and D) compared with controls, observed by NMR analysis of the hepatic tissue (Figure 3D and Supplemental Figure 8D) and confirmed by Oil Red O staining of liver slices (Figure 3E). Collectively, these data suggest that the absence of Endo1 results in a redistribution of lipids between organs with increased lipid uptake by adipose tissues (mainly s.c. and gonadal). One of the regulatory mechanisms is a modulation of CD36 levels at the surface of adipocytes, thereby adjusting the distribution and storage of fat throughout the body and preventing the accumulation of ectopic fat in other tissues, such as the liver.

### Endo1 deletion decreases systemic and tissue inflammation.

**Figure 4:**
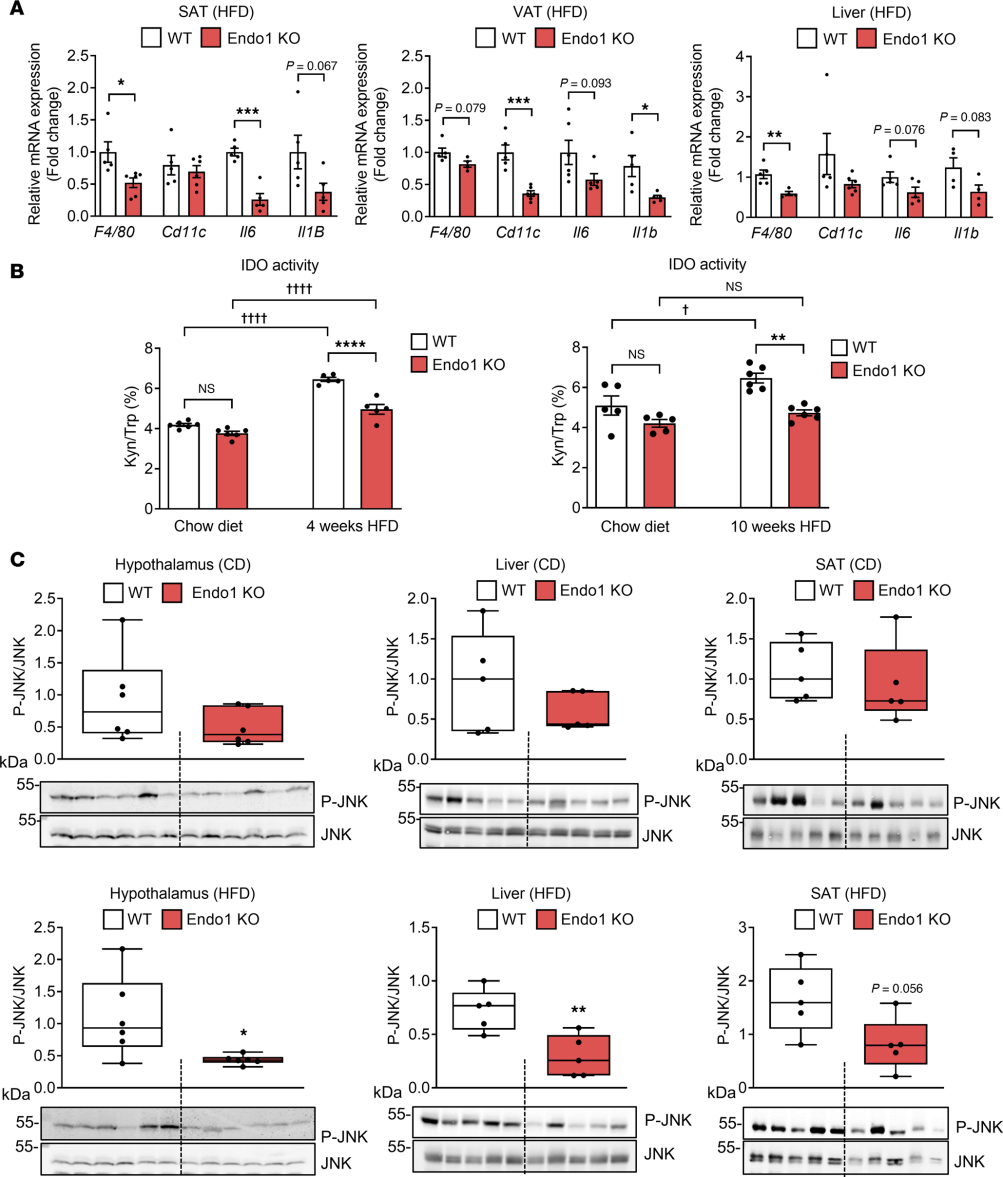
Absence of Endo1 decreases HFD-induced low-grade inflammation. (**A**) Transcripts of inflammatory markers, determined by qPCR, in the visceral adipose tissue (VAT), s.c. adipose tissue (SAT), and liver of mice fed a HFD for 4 weeks. Results are expressed as mean ± SEM (*n* = 5–6). *P < 0.05; **P < 0.01; ***P < 0.005 versus WT. Two-tailed *t* test. (**B**) Indoleamine 2,3-dioxygenase (IDO) activity in plasma of mice fed a HFD for 4 or 10 weeks. Results are expressed as mean ± SEM (*n* = 5–6). **P < 0.01; ****P < 0.001 versus WT. ^†^*P* < 0.05; ^††††^*P* < 0.001 versus CD. One-way ANOVA with Tukey’s test. (**C**) Immunodetection of phosphorylated JNK in hypothalamus, liver, and s.c. tissue from mice fed a CD or a HFD for 4 weeks. Each lane corresponds to an individual mouse. Results are expressed as mean ± SEM (*n* = 5–6). *P < 0.05; **P < 0.01 versus WT. Two-tailed *t* test. The molecular weights of protein markers are indicated (kDa).

The phenotype observed in obese Endo1-KO mice suggests a healthy adipose tissue phenotype, despite the HFD, ameliorating the pernicious effects of the obesogenic diet. Since one of the main aspects of pathological obesity is local and systemic low-grade inflammation development (22), we assessed the inflammatory status of Endo1-KO mice on HFD. Endo1-KO mice showed a decrease in the expression of the inflammatory cytokines (*IL1B* and *IL6*) as well as a decrease in markers of infiltrating macrophages (*F4/80*, *CD11C*) in the liver and adipose tissue (s.c. and visceral) of animals fed a HFD (Figure 4A). These data indicate that increased lipid uptake in adipose tissue and decreased steatosis are accompanied by an attenuated inflammatory state in Endo1-KO mice on HFD. Corroborating the observation of less inflammatory response in HFD compared with obese WT mice, Endo1-KO mice on HFD showed a significant drop in the activity of the immunomodulatory enzyme indoleamine 2,3-dioxygenase (IDO), which is a key enzyme involved in the degradation of tryptophan (TRP) metabolism to kynurenine (KYN), and the activity of which is strongly induced by proinflammatory cytokines in obesity (23) (Figure 4B). The decrease in the Kyn/Trp ratio in Endo1 KO in HFD, related to IDO activity, reveals attenuated systemic inflammation in obese Endo1-KO mice. In addition, chronic activation of JNK is a marker of tissue inflammation due to its strong activation in response to increased inflammatory cytokines (24). Consistent with a decrease in systemic inflammation in Endo1-KO mice, Endo1 deletion is associated with a significant decrease in JNK phosphorylation in the hypothalamus and liver and is associated with no significant changes of p-JNK in the adipose tissue (Figure 4C), suggesting an overall reduction in obesity-related central and peripheral inflammation in Endo1-KO mice.

### Endo1-KO mice are protected from obesity-induced impairment of glucose homeostasis.

**Figure 5:**
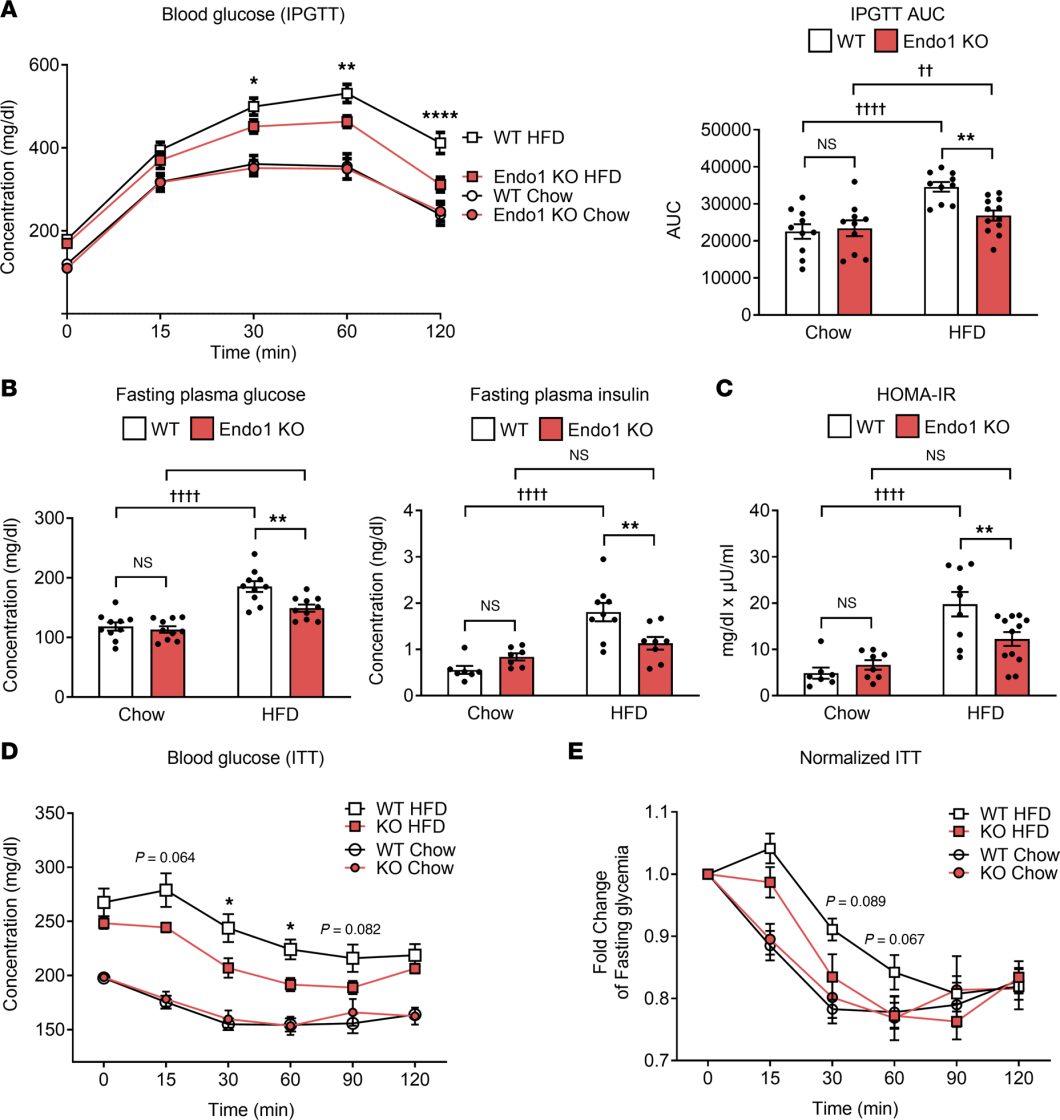
Absence of Endo1 correlates with improved glucose homeostasis in obese mice. (**A**) I.p. glucose tolerance test (IPGTT) after a 4-week HFD. Results are expressed as mean ± SEM (*n* ≥ 8). *P < 0.05; **P < 0.01; ****P < 0.001 versus WT. Two-way ANOVA with Bonferroni correction. AUC of the IPGTT (right panel). **P < 0.01 versus WT. ^††^*P* < 0.01; ^††††^*P* < 0.001 versus CD. One-way ANOVA with Bonferroni correction. (**B**) Plasma glucose and insulin levels after 16 hours of fasting. Results are expressed as mean ± SEM (*n* ≥ 7). **P < 0.01 versus WT; ^††††^*P* < 0.001 versus CD. One-way ANOVA with Bonferroni correction. (**C**) Assessment of the homeostatic model of insulin resistance (HOMA-IR). Results are expressed as mean ± SEM (*n* ≥ 7). **P < 0.01 versus WT; ^††††^*P* < 0.001 versus CD. One-way ANOVA with Bonferroni correction. (**D**) Insulin tolerance test (ITT) after 6-hour fasting on mice fed a 12-week HFD or CD. Results are expressed as mean ± SEM (*n* ≥ 8). *P < 0.05 versus WT. Two-way ANOVA with 2-stage linear step-up procedure of Benjamini, Krieger, and Yekutieli correction. (**E**) Blood glucose levels during ITT normalized with fasting glycemia. Results are expressed as mean ± SEM (*n* ≥ 8). Two-way ANOVA with 2-stage linear step-up procedure of Benjamini, Krieger, and Yekutieli correction.

We then investigated whether the attenuation of chronic inflammation observed in Endo1-KO mice could improve glucose homeostasis. Endo1-KO animals fed a CD have a similar glycemic profile to WT mice (Supplemental Figure 9 and Figure 5, A–C). However, as early as 4 weeks on HFD, WT control mice developed GI as expected, whereas glucose tolerance was still preserved and normal in Endo1-KO mice despite the HFD (Supplemental Figure 9A), suggesting a maintenance of glucose homeostasis in Endo1-KO mice at the early stage of HFD. After 10 weeks on HFD, while both Endo1-KO and WT mice on HFD showed similar glucose-induced insulin secretion (Supplemental Figure 9B), Endo1-KO mice developed mild GI compared with mice on CD, but this intolerance was lower than in WT mice on HFD, which instead showed pronounced intolerance (Figure 5, A and B). The improved glucose tolerance of Endo1-KO mice compared with WT mice after 10 weeks on HFD was accompanied by a decrease in fasting blood glucose and insulin levels, resulting in a lower insulin resistance index (HOMA-IR) in Endo1-KO mice (Figure 5C). We observed that, on HFD, Endo1-KO mice tended to respond more efficiently than WT mice to exogenous insulin during insulin tolerance test (ITT), suggesting a state of lower insulin resistance (Figure 5, D and E). In accordance with this, compared with WT mice, Endo1-KO showed improvements in metabolic parameters, such as a reduction in cholesterol and plasma β-hydroxybutyrate (BHB) in KO mice (Supplemental Figure 9C), where lower levels of BHB — indicating improved glucose utilization — provide additional support of ameliorated insulin action. In addition, since there is a positive correlation between CD36 function and insulin sensitivity at the adipose tissue and adipocyte levels (25–27), we evaluated and observed a higher insulin signaling in adipocytes isolated from Endo1-KO mice compared with WT controls (Supplemental Figure 9D). Similarly, increased endogenous AKT phosphorylation in SAT from Endo1-KO mice was also detected compared with WT controls under HFD, suggesting higher insulin sensitivity in Endo1-KO adipose tissues (Supplemental Figure 9E). These data evoke that deletion of Endo1 in mice promotes protective conditions against impaired glucose homeostasis, decreasing the development of T2D at early and later stages of HFD.

### Low LEPROT expression correlates with a better metabolic profile in humans.

**Figure 6:**
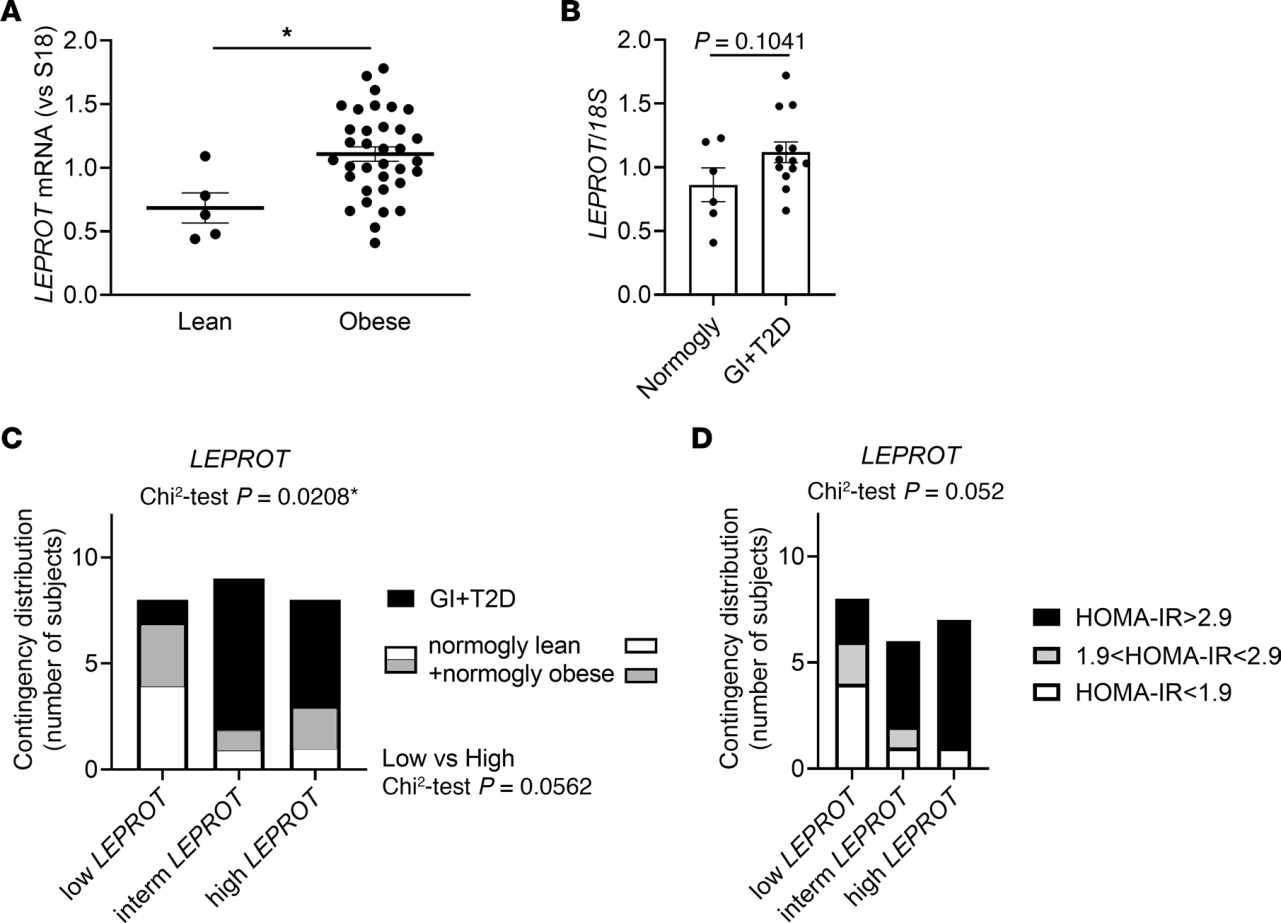
Low levels of Endo1 correlate with improved glucose homeostasis in obese humans. (**A**) *LEPROT* transcript levels in humans with obesity versus lean humans. Results are expressed as mean ± SEM (*n* ≥ 5). *P < 0.05 versus humans with obesity. Two-tailed *t* test. (**B**) *LEPROT* mRNA levels in patients with obesity and with impaired glucose tolerance (GI) or type 2 diabetes (T2D) compared with normoglycemic patients with obesity (18 years < age < 55 years; 36 < BMI < 49). Results are expressed as mean ± SEM (*n* ≥ 6). Two-tailed *t* test. (**C**) Contingency distribution of lean individuals and patients with obesity according to low, medium, or high *LEPROT* mRNA levels classified by tertiles. A χ^2^ test was used. (**D**) Contingency distribution of patients with obesity according to low, intermediate, or high *LEPROT* levels and grouped into 3 HOMA-IR index categories. The χ^2^ test between low and high *LEPROT* gives a *P* value of 0.052.

In humans, GEO2R analysis of the Gene Expression Omnibus (GEO) GSE2508 (GPL92) data set from the study performed by Lee et al. (28) shows that adipocytes isolated from male and female Pima Indian patients with severe obesity (BMI = 55 ± 8; *n* = 19) are characterized by increased inflammation and a higher level of *LEPROT* mRNA compared with nonobese controls (BMI = 25 ± 3; *n* = 20), confirming the association between *LEPROT* expression and human obesity at the level of adipocytes (log [fold change] = 0.432; *P* = 4.67 × 10^–5^; Supplemental Figure 10A). Interestingly, GEO2R analysis of GEO GSE27951 data set (from the study; ref. 29) reveals that, among patients with a BMI characterized as overweight (25 < BMI < 30) and those with obesity (30 < BMI < 40), *LEPROT* levels are upregulated (log [fold change] = 0.614; *P* = 0.0354) in the SAT of patients with glucose intolerance and T2D (25 < BMI < 40; mean BMI = 33.5; *n* = 16) compared with patients with normoglycemia who are overweight or are diagnosed with obesity (25 < BMI < 40; mean BMI = 33.3; *n* = 8). Similarly, in our own study investigating human tissues, we observed that *LEPROT* transcripts are increased in the adipose tissue of patients with obesity compared with lean control patients (Figure 6A). We detected that individuals with obesity and GI or T2D are characterized by elevated plasma levels of HbA1C and the inflammatory cytokine IL-6, by lower plasma levels of adiponectin (Supplemental Figure 10B), and by higher levels of *LEPROT* in their adipose tissue (Figure 6B). The contingency distribution of lean and obese individuals according to low, intermediate, or high *LEPROT* mRNA levels classified by tertiles, thus, reveals a greater representation of individuals with intermediate or high *LEPROT* levels in glucose-intolerant or diabetic patients (χ^2^ test, *P* = 0.0208) (Figure 6C). We further divided obese individuals into low, intermediate, and high *LEPROT* levels and grouped them into 3 HOMA-IR categories assessing normal insulin sensitivity (HOMA-IR < 1.9), intermediate insulin resistance (1.9 < HOMA-IR < 2.9), or insulin resistance status (HOMA-IR > 2.9) (Figure 6D). This contingency distribution tends to show a greater number of patients with insulin resistance when their *LEPROT* level is intermediate or high compared with those with the lowest *LEPROT* level (χ^2^ test, *P* = 0.052) (Figure 6D). In the latter category of individuals with low *LEPROT* levels, a majority of them show better insulin sensitivity. These data suggest that low levels of *LEPROT* in humans with obesity and its deletion in obese mice are associated with an obesity accompanied by attenuated GI or T2D.

## Discussion

Cell surface receptors are essential for proper signaling across the cell, usually binding to ligands that cannot cross the plasma membrane to activate a variety of signaling cascades that lead to subsequent cellular response, control of cell metabolism, production and release of secreted signals, or regulation of cellular proliferation or apoptotic processes. Therefore, their presence at the cell surface must be strictly regulated to avoid excessive or insufficient response to extracellular signals by modulating their trafficking to and from the plasma membrane and their degradation/recycling. Among the regulators of receptor trafficking, Endo1 appears as a key regulator of surface levels of the leptin receptor (15), growth hormone receptor (14), and CD36 (shown in this study). Endo1 is a highly conserved vesicular trafficking protein capable of controlling the presence of the leptin receptor at the cell surface by trapping it inside the cell and promoting its trafficking from endosomes to lysosomes for degradation (7, 12, 13). The action of Endo1 has also been linked to the growth hormone receptor by reducing its expression and sensitivity to the growth hormone in Endo1 and Endo2 overexpressing transgenic mice (14). Here we show that Endo1 also regulates CD36, a multiligand receptor involved in different metabolic processes, by modulating its localization on the cell surface of adipocytes, revealing that Endo1 is an important intracellular regulator of metabolic receptors.

Endo1 is expressed in the hypothalamus, as well as in peripheral metabolic tissues. The importance of the tissue target in which Endo1 exerts its functions is fundamental to assess and understand its biological significance. In our previous work, we studied the modulation of Endo1 at the central level, showing that its specific silencing in the ARH of mice has beneficial effects after a HFD by improving leptin sensitivity and reducing body weight, accompanied by an improvement in blood lipid parameters and liver steatosis (16). This is associated with a modest effect on glucose tolerance when Endo1 silencing is conducted prior to the initiation of HFD (17). However, ablation of Endo1 in the ARH after a prolonged HFD contributes to the alteration of glucose homeostasis by a reduction in leptin-mediated PI3K/AKT signaling (17).

In the current study, the global and complete deletion of Endo1 produces a different phenotype under HFD, likely driven by Endo1/CD36 in adipose tissues. The latter would contribute to improving the overall inflammatory state and glucose homeostasis while facilitating lipid accumulation in adipose tissue. Of note, the silencing of Endo1 in the ARH (Endo1^shRNA^ ^ARH^), under HFD, is not comparable with the complete deletion of Endo1 (Endo1-KO) under HFD. The HFD condition exposes the body to a harmful lipid load that goes beyond the function of LepR. However, in a context of reduced lipid load, our earlier investigations involving Endo1^shRNA^ ^ARH^ mice (prior to HFD) and the current study with Endo1-KO mice yield consistent results. Indeed, parallels can be drawn between the context of “Endo-1-KO on HFD” (as in the current study) and “Endo1^shRNA^ ^ARH^ mice followed by HFD and a diet switch from HFD to CD” (16). In both cases, there is a reduction in excessive organ exposure to lipid loads, achieved either by transitioning from HFD to CD (16, 17) or by promoting fat accumulation in adipose tissue (seen in the current study). Both approaches result in lower levels of steatosis in Endo1 KO or Endo1^shRNA ARH^ compared with WT controls (16). Both scenarios alleviate and limit GI.

CD36 is a multiligand receptor with multiple functions ranging from increasing lipid uptake to lipid metabolism (30). The pathophysiological role of CD36 is complex, and the metabolic regulation of CD36 can be beneficial or detrimental depending on the tissue context or conditions. For example, global KO models of CD36 are resistant to carbohydrate-induced hepatic steatosis (31) and have altered blood lipid parameters (32). Its deletion in brown adipose tissue results in impaired thermogenesis (33) and its absence in lymphatic endothelial cells to obesity and insulin resistance (34). Inversely, its overexpression in muscle reverses insulin resistance and diabetes (35), and its role in the liver remains controversial (36, 37). Studies on CD36 in adipose tissues have generally focused on its ability to increase lipid uptake and lipid metabolism, with a general consensus on its functions. Here, the increase in adipocyte surface level of CD36 by the deletion of Endo1, promoting a beneficial activity of CD36 on adipose tissue lipid accumulation, is consistent with the previously described effects of CD36 upregulation in adipose tissue with increased capacity for lipid uptake. In humans, CD36 deficiency has been associated with insulin resistance (38), and human CD36 deficiency or human CD36 variants have been associated with abnormal plasma lipid levels (39–41). More recently, in human adipose tissue, the extent of CD36 surface abundance in adipocytes has been correlated with a better function or dysfunction of adipose tissue, depending on energy status (42). Consistent with this, the increased expression of Endo1 in the white adipose tissue (WAT) during HFD would likely lead to an imbalance in the localization of CD36 on the surface of adipocytes in obesity, contributing to adipose tissue dysfunction. In addition, in contrast to CD36^–^ primary adipocyte precursors in the SAT, expression of CD36 in adipocyte precursors is associated with reduced metabolic risk and increased lipid uptake capacity, leading to increased adipogenesis (43). This observation is consistent with our results on the improved metabolic profile resulting from higher levels of CD36 on the adipocyte plasma membrane and the subsequent increase in lipid uptake by adipocytes in the absence of Endo1. Interestingly, we observed that Endo1 expression is higher in “beneficial”adipose tissue depots — i.e., SAT — than in VAT, the accumulation of which may be detrimental, suggesting that, under Endo1 KO conditions, CD36 would be predominantly upregulated at the surface of SAT adipocytes to preferentially accumulate lipids. We thus define here that Endo1 is a potentially novel regulator of CD36 that affects the localization of CD36 in adipocytes and lipid uptake function.

Our study has potential limitations, as we did not directly assess whether the presence of Endo1 in adipose tissue and its effect on CD36 are the primary cause of the observed phenotype. Tissue-specific deletion of Endo1 would help clarify this aspect. Nevertheless, several compelling pieces of evidence underscore the significance of Endo1 and CD36 in adipose tissue, including (a) the predominant expression of both Endo1 and CD36 in adipose tissue compared with other organs; (b) the significant increase in CD36 surface localization and its functional role in isolated Endo1-KO adipocytes; (c) the relatively moderate effect of Endo1 on the leptin system in the hypothalamus, particularly due to its opposing regulation of 2 LEPR signaling pathways; and (d) the consistency between the current data and the results of our previous studies, both of which are based on the limitation of lipid loads, here resulting from a preferential lipid accumulation in adipose tissues via CD36. Overall, we conclude that the concomitant effect of Endo1 on hypothalamic LEPR function, leading to a reduction in food intake through the enhanced STAT3 pathway, along with its effect on CD36 facilitating fat accumulation in adipose tissue during HFD likely contribute to the observed metabolic phenotype in Endo1-KO mice.

The phenotype of the Endo1-KO mouse model is in agreement with human observations where the transcript level of Endo1 is found increased in the adipose tissue of patients with obesity, while a lower level of Endo1 in the adipose tissue is rather favorable to a healthier metabolic status in these patients. A study of Endo1 in human obesity in a larger cohort will help to confirm our current observations. The Endo1-KO mouse model shares, therefore, characteristics similar to those of individuals with MHO. The concept of MHO refers to a condition in which a small proportion of the obese population does not exhibit the pernicious effects associated with altered body weight, such as dyslipidemia, insulin resistance and systemic inflammation (44). The MHO phenotype in humans includes preserved insulin-sensitive glucose homeostasis and a reduced inflammatory profile in adipose tissue (45) with healthy adipose tissue expansion (2). MHO has also been reported in several mouse models showing a phenotype with a normal or better metabolic profile than their WT obese counterparts (46–51). For example, deletion of the G protein–coupled receptor LPA4 showed a MHO phenotype with WAT expansion and protection from WAT inflammation, steatosis, and insulin resistance (50). However, in humans, MHO most likely represents a transient phenotype where the risk of developing metabolic disorders and cardiometabolic diseases is reduced compared with metabolically unhealthy obesity but remains higher than in healthy lean individuals (3). The absence of obesity-related symptoms at one time point does not necessarily protect against their occurrence at a later time (52).

In summary, Endo1 is a key regulator of lipid and glucose metabolism in mice through its regulation of metabolic receptors such as CD36. The absence of Endo1 in mice and the decrease in its expression in human adipose tissue are associated with a phenotype with healthier metabolic condition through the decrease of insulin resistance and steatosis as well as a lower inflammatory state.

## Methods

### Sex as a biological variable.

Preliminary studies with male and female KO mice were conducted simultaneously, revealing a similar phenotype; thus, sex was not considered as a biological variable in this study.

### Animal work.

C57BL/6N male mice (Janvier) were housed in specific pathogen–free biosafety level 2 animal facility in a standard 12-hour on/off light cycle, according to the guidelines approved by the institutional research animal committee (approval B 75-14-02). Mice were fed a standard rodent diet or 45% kcal HFD (D12451; Research Diets) and were provided with water and food ad libitum. Body weight was monitored weekly.

### Generation of Endo1-KO mice.

Endo1-KO mice were generated as described by Vauthier et al. (17). Unlike humans, the mouse Endo1 gene, located on chromosome 4, contains 4 exons and is not genetically linked to the LEPR gene, allowing for the generation of mice specifically deleted of the Endo1 gene without affecting LEPR expression. Mice floxed for Endo1 were generated using a targeting vector produced by the NIH Knockout Mouse Project (KOMP), in which exon 2 is flanked by loxP sites and introduced by homologous recombination into 129/SV CK35 embryonic stem cells. The neomycin resistance cassette flanked by FRT sites was excised by crossing Endo1 lox/wt mice with mice expressing FLP. Mice with an Endo1 deletion were generated by crossing C57BL/6 mice expressing the Cre recombinase under the control of the ubiquitous mouse EIIa promoter (EIIa-Cre mice) with Endo1-floxed mice. Disruption of the Endo1 gene was assessed by PCR from DNA extract, and the absence of Endo1 protein was confirmed by Western blot.

### Fasting and refeeding experiments.

WT and Endo1-KO C57BL/6N male mice were individually housed and fasted just before the dark phase for 16 hours. After an overnight fasting period, mice were given a standard diet for 2 or 6 hours, and food intake was measured. For leptin challenge, 16-hour–fasted mice were i.p. injected with human leptin (1 mg/kg in PBS, Protein Laboratories Rehovot Ltd. [PLR]) or with control buffer.

### Indirect calorimetry.

Six-month-old mice (12 Endo1-KO and 12 WT controls) either fed with a HFD (*n* = 6 per group) or a conventional diet group (CD, *n* = 6 per group) were individually housed in metabolic cages 7 days before experimental measurements, at 22°C ± 1°C room temperature (RT) with their respective regimen and water available ad libitum. Metabolic exploration was performed as described previously (53) using indirect calorimetric cages (Labmaster, TSE Systems GmbH). Each set of measurement represents 4 consecutive days. Mice were analyzed for whole energy expenditure (kcal/hr), O_2_ consumption, CO_2_ production, RER (VCO_2_/VO_2_), food intake (g), and locomotors activity (beambreak/hour) using calorimetric cages with bedding, food, and water ad libitum (Labmaster, TSE Systems GmbH). The flow was calibrated with a O_2_ and CO_2_ mixture of known concentration (Air Liquide). Whole energy expenditure was calculated according to the Weir equation (54). Fatty acid oxidation was calculated from the following equation: fatty acid oxidation (kcal/hr) = energy expenditure X (1-RER/0.3) according to Bruss et al. (55) Estimation of resting metabolism was made according to Péterfi et al. (53). Mice were monitored for body weight and composition at the entry and at the exit of the experiment. Body mass composition (lean body mass, fat mass, free water, and total water content) was analyzed using an Echo Medical systems’ EchoMRI (Whole Body Composition Analyzers, EchoMRI), according to manufacturer’s instructions. Data collection was recorded every 15 minutes during the whole experiments, and data extracted were raw values of VO_2_ consumed, VCO_2_ produced (mL/h) and energy expended (kcal/h). Since extracted values were uncorrelated to any body mass compartment (56), each value was normalized either by total body weight or whole lean body mass extracted from the EchoMRI analysis.

### Tissue extraction and weight measurements.

All tissues were dissected directly after each treatment and immediately frozen in dry ice or liquid nitrogen. After extraction, tissues were processed for RNA/protein extraction or stored at –80°C. Liver, VAT (located inside the peritoneal cavity around the small intestine), GAT (attached to the epididymis and testis) and SAT (in the hips under the skin) were totally extracted to measure their weight.

### Liver composition analysis.

Liver fat and lean content was determined by using a magnet NMR minispec mq60 (Bruker Corporation). For detection of neutral lipids, liver cryosections were fixed and stained with the Oil Red O technique using 0.23% dye dissolved in 65% isopropyl alcohol for 10 minutes as previously described (57).

### Hormonal levels determination in plasma.

TRP and KYN rates were measured from plasma by high-performance liquid chromatography (HPLC) by isocratic LC with coulometric detection (58). The KYN/TRP ratio, calculated from absolute concentrations of KYN and TRP, was used as an index of IDO activity. Leptin levels were measured by AlphaLISA (Perkin Elmer) or Ultra Mouse Leptin ELISA Kit (Crystal Chem), and Insulin levels were measured by Ultra Mouse Insulin ELISA Kit (Crystal Chem). Fasting plasma C-peptide, insulin, glucagon, Ghrelin, GLP-1, and Amylin were measured with MILLIPLEX Mouse Metabolic Hormone Multiplex Assay (Merck Millipore). Blood samples for GLP-1 measurements were treated with the DPP-4 inhibitor (Merck Millipore) immediately after collection of blood.

### Adipocyte isolation and differentiation into white adipocytes.

Adipocytes from the s.c. fat pad were isolated and washed 3 times with PBS supplemented with a 1% penicillin/streptomycin cocktail (Thermo Fisher Scientific). The tissue was mechanically cut into small pieces and resuspended in 0.2% collagenase type I (Invitrogen) in DMEM and incubated at 37°C for 90 minutes. After 5 minutes of centrifugation at 400*g* (room temperature), 2 fractions were collected. The top fraction, corresponding to the mature adipocytes, was used immediately or frozen at –80°C for further analysis; the pellet fraction, corresponding to the adipose-derived stromal cells, was plated in Petri dishes coated with Poly-L-lysine (Sigma-Aldrich) and placed in a 5% CO_2_ cell incubator for differentiation studies (59). Adipocytes were differentiated from the preadipocytes present in the stromal fraction as described previously (60); adipocyte differentiation rate was monitored by Oil Red O lipid staining (Sigma-Aldrich).

### Myocytes isolation and differentiation.

Myoblast isolation and differentiation to myotubes was performed as described by Hindi et al. (61). Briefly, myoblast from mouse gastrocnemius muscles were isolated and plated in petri dishes coated with 10% ECL matrix (Merck Millipore) and incubated in DMEM supplemented with 2% FBS for 4 days in a 5% CO_2_ cell incubator until large and mature myotubes could be clearly seen.

### Immunoblot.

Whole tissues were lysated with a TissueLyser II (Qiagen Retsch GmbH) in RIPA buffer (200 mM Tris-HCl [pH 7.4], 130 mM NaCl, 10% [v/v] glycerol, 0.1%, 1% [v/v] Triton X-100, 10 mM MgCl_2_) with phosphatase and protease inhibitors (Sigma-Aldrich). After 30 minutes of centrifugation at 12,000*g* at 4°C, clear lysates were quantified (RC DC Protein Assay, Bio-Rad) and equal amount of proteins were prepared in 1× Laemmli buffer (62.5 mM Tris-HCl [pH 6.8], 2% SDS, 5% [v/v] glycerol, 0.01% bromophenol blue) for intracellular proteins, and in 2× Laemmli buffer (125 mM Tris-HCl [pH 6.8], 10% SDS, 5% [v/v] glycerol, 0.01% bromophenol blue) for membrane proteins. For cell lines and primary cultures, lysates were obtained by mild sonication directly in 1× or 2× Laemmli buffer. Protein extracts from whole tissues and cell lysates were separated by sodium dodecyl sulfate–polyacrylamide gel electrophoresis, transferred to nitrocellulose membranes, and blotted with the selected antibodies. Immunoblots were scanned on the Odyssey infrared Imaging System (LI-COR Biosciences GmbH) and analyzed with ImageJ software (NIH). Primary Endo1 polyclonal rabbit antibody was obtained as described previously (13); primary phospho-STAT3 (catalog 9145S), phospho-AKT (catalog 4060S), and phospho-JNK (catalog 4668S) polyclonal rabbit antibodies as well as β-actin (catalog 3700S), α-tubulin (catalog 3873S), total STAT3 (catalog 9139S), total AKT (catalog 2920S), and total JNK (catalog 3708S) polyclonal mouse antibodies were purchased from Cell Signaling; primary FLAG mouse antibody (catalog B3111) was purchased from Sigma-Aldrich; primary c-Myc mouse antibody (catalog M4439) was purchased from Santa Cruz Biotechnology Inc.; primary leptin antibody (catalog ab16227) was purchased from Abcam; and primary CD36 goat antibody (catalog AF2519) was purchased from R&D Systems.

### Endo1 immunostaining in mouse hypothalamic brain slices.

Fresh brains were immediately frozen in OCT after dissection and cut on Leica CM3050S cryostat at 14 μm. Sections were fixed 10 minutes with 4% PFA at RT and treated for 1 hour with blocking buffer containing 5% donkey serum and 0.3% Triton X-100 in PBS before incubation with primary antibody (Endo1 antibody, 1/750 in 0.3% Triton X-100 in PBS) overnight at 4°C. Sections were rinsed 3 times 10 minutes in PBS at RT before incubation for 2 hours with donkey anti–rabbit Alexa 568 (Invitrogen, A10042, 1/1,500 in 0.3% Triton X-100 in PBS). After 3 washes of 10 minutes with PBS, sections were counterstained 5 minutes with DAPI (1/5,000 in PBS, 5 mg/mL), washed, and mounted in Mowiol (Sigma-Aldrich). Images were taken by a LSM 710 confocal microscope.

### qPCR.

Relative mRNA levels were quantified with quantitative PCR (qPCR) using fluorescent TaqMan technology. Total RNA was isolated from tissues using Trizol (Invitrogen) according to the manufacturer’s recommendations or using RNeasy Micro Kit (Qiagen) for dissected hypothalamus. Reverse transcription was performed using Maxima First Strand cDNA Synthesis for RT-PCR (Thermo Fisher Scientific). TaqMan inventoried gene expression assays (Invitrogen) were used to analyze gene expression: Mm00838516 (*Leprot*), Mm00475829_g1 (*Agrp*), Mm01410146_m1 (*Npy*), Mm00435874_m1 (*Pomc*), Mm00434228 (*Il1b*), Mm00446190 (*Il6*), Mm00802529 (*F4/80*), and Mm00498701 (*Cd11c*). *Rplp0* (Mm00725448) was used as a housekeeping gene; results are expressed as arbitrary units relative to the average value of the control.

### Glucose tolerance measurement and ITT.

Glucose-tolerance tests (GTT) were performed by i.p. D-glucose (Sigma-Aldrich, 2 g/kg) injection following a 16-hour fast. ITT was performed by i.p. injection of insulin (I2643, MilliporeSigma) (0.5 IU/kg) following a 6-hour fast. Blood glucose levels were determined from the tail vein at 0, 15, 30, 60, 90, 120, and 180 minutes using a hand-held glucometer (One Touch Ultra).

### Homeostatic model assessment for insulin resistance (HOMA-IR).

HOMA-IR index was calculated from the values of fasting serum glucose (mg/dL) and fasting serum insulin (μU/mL) by using the following formula: HOMA-IR = fasting glucose value (mg/dL) × fasting insulin value (μU/mL)/405 (62). Lower values than the control were considered as an indication of better insulin sensitivity.

### Lipid uptake.

Quantification of lipid uptake in cells was performed using the Free Fatty Acid Uptake Assay Kit (Abcam) according to the manufacturer’s recommendations. Cells treated with 150 nM human insulin (Sigma-Aldrich) were used as a positive control.

### Cell surface biotinylation.

Cell surface proteins were isolated by using the Pierce Cell Surface Protein Isolation kit (Thermo Fisher Scientific) according to the manufacturer’s recommendations.

### Immunofluorescence microscopy.

Preadipocytes from the adipose-stromal fraction were plated onto glass coverslips coated with Poly-L-lysine (Sigma-Aldrich) and differentiated to mature adipocytes. Cells were fixed with 4% PFA (PolySciences Inc.) for 15 minutes at RT. Fixed cells were permeabilized with 0.3% saponin for 10 minutes and blocked in PBS containing 10% horse serum for 1 hour at RT. Next, cells were incubated with primary Endo1 (1:500) (generated as described previously; ref. 13) and primary CD36 (1:1,000) (RD Systems, catalog AF2519), primary Endo1 (1:500) and primary 53K (1:1,000, Abcam, catalog ab27043), or primary GM130 (1:1,000, Abcam, catalog ab169276) antibodies overnight at 4°C. Alexa Fluor 488– and/or Cy3-labeled conjugated secondary antibodies were used for visualization. Confocal microscopy was performed with an SP2 confocal laser-scanning microscope (Leica) using a ×100/1.4 numerical aperture oil immersion lens. Double label immunofluorescence signals were sequentially collected using single fluorescence excitation and acquisition settings to avoid cross-over. Images were processed using ImageJ software (NIH). To isolate the signal from cell surface proteins, a permeabilization step was completely avoided and fluorescence intensity was quantified by calculating the corrected total cell fluorescence per area (CTCF/area) using ImageJ software (63)

### Cell culture and transfection.

Human Embryonic Kidney (HEK) 293T cells from LGC Standards were grown in DMEM supplemented with 10% (v/v) FBS, 4.5 g/L glucose, and 1 mM glutamine (Invitrogen). cDNAs for Endo1 (6Myc-Endo1) and CD36 (FLAG-huCD36) were transfected in cells with JetPEI (Polyplus transfection) according to the manufacturer’s instructions.

### Co-IP.

Transfected cells or fresh tissues were lysed in TEM buffer (25 mM Tris-HCl [pH 7.5], 2 mM EDTA, 10 mM MgCl_2_, and protease and phosphatase inhibitors), supplemented with 0.5% (v/v) Triton X-100, and incubated on an orbital rotator at 4°C for 4 hours. Lysates were cleared by centrifugation at 12,000*g* for 1 hour at 4°C and immediately subjected to Endo1 or CD36 immunoprecipitation overnight on an orbital rotator at 4°C, by adding 2 μg of primary antibody as specified for each experiment. Protein G Sepharose beads (Sigma-Aldrich), previously saturated with 2% BSA, were then added for 2 hours on an orbital rotator at 4°C, followed by 4 washes with lysis buffer. Protein complexes were denatured in 2% Laemmli buffer supplemented with 50 mM DTT and analyzed by immunoblot.

### Human samples.

Human adipose tissue samples were obtained from a total of 35 patients with severe obesity candidates for bariatric surgery who were recruited in the Nutrition Department, Pitié-Salpêtrière Hospital, and they were operated in the Surgery Departments of Hôtel-Dieu, Ambroise Paré and Pitié-Salpêtrière hospitals between 2007 and 2016.

### Public databases.

mRNA expression of LEPROT and CD36 from different human tissues and single-cell analysis were obtained and analyzed from the Human Protein Atlas (20); data are accessible via https://www.proteinatlas.org/about/download mRNA expression patterns of CD36 and LEPROT genes in human adipose tissue from single-cell RNA-Seq and the correlation between CD36 and LEPROT in human adipocytes from single-cell analysis were obtained from ref. 21. Single-cell RNA expression and count data were analyzed from the Single Cell Portal (study no. SCP1376).

### Statistics.

Data were analyzed by 2-tailed unpaired Student’s *t* test or by 1-way or 2-way ANOVA (corrected for repeated measures, if required) followed by Bonferroni; Tukey’s multiple-comparison tests; Benjamini, Krieger, and Yekutieli correction; or Šídák’s multiple-comparison test as specified, using Graphpad Prism 8 software. Results are presented as mean ± SD or SEM. *P* < 0.05 was considered statistically significant.

### Study approval.

All animal experimental procedures were approved by the French ethics committee (no. 2017122811477356 – V4 APAFiS #16771). Ethical approval for human samples was obtained from the Research Ethics Committee of Hôtel-Dieu Hospital (CPP Ile-de-France No.1). Informed written consent was obtained from all patients, and the protocol was registered on www.clinicaltrials.gov (NCT01655017). Detailed information can be retrieved at ref. 64.

### Data availability.

Data values associated with this study are reported in the Supporting Data Values file.

## Author contributions

Conceptualization was contributed by ARR and JD. Investigation was contributed by ARR, RGPD, QZ, CR, JML, CCG, VM, KC, and JD. Generation of animal model was contributed by MDC. Resources were contributed by YR. Writing of the original draft was contributed by ARR and JD. Review and editing was done by all authors. Funding acquisition was contributed by ARR, RJ, and JD. Supervision was contributed by ARR and JD.

## Supplementary Material

Supplemental data

Unedited blot and gel images

Supporting data values

## Version 1 05/08/2024

Electronic publication

## References

[1] April-Sanders AK, Rodriguez CJ (2021). Metabolically healthy obesity redefined. JAMA Netw Open.

[2] Vishvanath L, Gupta RK (2019). Contribution of adipogenesis to healthy adipose tissue expansion in obesity. J Clin Invest.

[3] Bluher M (2020). Metabolically healthy obesity. Endocr Rev.

[4] Friedman JM (2019). Leptin and the endocrine control of energy balance. Nat Metab.

[5] Pan WW (2018). Leptin and the maintenance of elevated body weight. Nat Rev Neurosci.

[6] Roujeau C (2014). New pharmacological perspectives for the leptin receptor in the treatment of obesity. Front Endocrinol (Lausanne).

[7] Roujeau C (2019). Endospanin 1 determines the balance of leptin-regulated hypothalamic functions. Neuroendocrinology.

[8] Cui H (2017). The cellular and molecular bases of leptin and ghrelin resistance in obesity. Nat Rev Endocrinol.

[9] Bailleul B (1997). The leptin receptor promoter controls expression of a second distinct protein. Nucleic Acids Res.

[10] Vauthier V (2012). Homozygous deletion of an 80 kb region comprising part of DNAJC6 and LEPR genes on chromosome 1P31.3 is associated with early onset obesity, mental retardation and epilepsy. Mol Genet Metab.

[11] Londraville RL (2017). On the molecular evolution of leptin, leptin receptor, and endospanin. Front Endocrinol (Lausanne).

[12] Belgareh-Touze N (2002). Yeast Vps55p, a functional homolog of human obesity receptor gene-related protein, is involved in late endosome to vacuole trafficking. Mol Biol Cell.

[13] Seron K (2011). Endospanins regulate a postinternalization step of the leptin receptor endocytic pathway. J Biol Chem.

[14] Touvier T (2009). LEPROT and LEPROTL1 cooperatively decrease hepatic growth hormone action in mice. J Clin Invest.

[15] Couturier C (2007). Silencing of OB-RGRP in mouse hypothalamic arcuate nucleus increases leptin receptor signaling and prevents diet-induced obesity. Proc Natl Acad Sci U S A.

[16] Vauthier V (2014). Endospanin 1 silencing in the hypothalamic arcuate nucleus contributes to sustained weight loss of high fat diet obese mice. Gene Ther.

[17] Vauthier V (2017). Endospanin1 affects oppositely body weight regulation and glucose homeostasis by differentially regulating central leptin signaling. Mol Metab.

[18] Nelms B (2017). A targeted RNAi screen identifies factors affecting diverse stages of receptor-mediated transcytosis. J Cell Biol.

[19] Abumrad NA (1993). Cloning of a rat adipocyte membrane protein implicated in binding or transport of long-chain fatty acids that is induced during preadipocyte differentiation. Homology with human CD36. J Biol Chem.

[20] Karlsson M (2021). A single-cell type transcriptomics map of human tissues. Sci Adv.

[21] Emont MP (2022). A single-cell atlas of human and mouse white adipose tissue. Nature.

[22] Rohm TV (2022). Inflammation in obesity, diabetes, and related disorders. Immunity.

[23] Brandacher G (2007). Chronic immune activation underlies morbid obesity: is IDO a key player?. Curr Drug Metab.

[24] Yung JHM, Giacca A (2020). Role of c-Jun N-terminal Kinase (JNK) in obesity and type 2 diabetes. Cells.

[25] Kontrova K (2007). CD36 regulates fatty acid composition and sensitivity to insulin in 3T3-L1 adipocytes. Physiol Res.

[26] Pietka TA (2014). Adipose and muscle tissue profile of CD36 transcripts in obese subjects highlights the role of CD36 in fatty acid homeostasis and insulin resistance. Diabetes Care.

[27] Yang P (2020). Loss of CD36 impairs hepatic insulin signaling by enhancing the interaction of PTP1B with IR. FASEB J.

[28] Lee YH (2005). Microarray profiling of isolated abdominal subcutaneous adipocytes from obese versus non-obese Pima Indians: increased expression of inflammation-related genes. Diabetologia.

[29] Keller P (2011). Gene-chip studies of adipogenesis-regulated microRNAs in mouse primary adipocytes and human obesity. BMC Endocr Disord.

[30] Pepino MY (2014). Structure-function of CD36 and importance of fatty acid signal transduction in fat metabolism. Annu Rev Nutr.

[31] Clugston RD (2014). CD36-deficient mice are resistant to alcohol- and high-carbohydrate-induced hepatic steatosis. J Lipid Res.

[32] Drover VA (2005). CD36 deficiency impairs intestinal lipid secretion and clearance of chylomicrons from the blood. J Clin Invest.

[33] Anderson CM (2015). Dependence of brown adipose tissue function on CD36-mediated coenzyme Q uptake. Cell Rep.

[34] Cifarelli V (2021). Visceral obesity and insulin resistance associate with CD36 deletion in lymphatic endothelial cells. Nat Commun.

[35] Heron-Milhavet L (2004). Muscle-specific overexpression of CD36 reverses the insulin resistance and diabetes of MKR mice. Endocrinology.

[36] Koonen DP (2007). Increased hepatic CD36 expression contributes to dyslipidemia associated with diet-induced obesity. Diabetes.

[37] Wilson CG (2016). Hepatocyte-specific disruption of CD36 attenuates fatty liver and improves insulin sensitivity in HFD-fed mice. Endocrinology.

[38] Miyaoka K (2001). CD36 deficiency associated with insulin resistance. Lancet.

[39] Yanai H (2000). Human CD36 deficiency is associated with elevation in low-density lipoprotein-cholesterol. Am J Med Genet.

[40] Kuwasako T (2003). Lipoprotein abnormalities in human genetic CD36 deficiency associated with insulin resistance and abnormal fatty acid metabolism. Diabetes Care.

[41] Ma X (2004). A common haplotype at the CD36 locus is associated with high free fatty acid levels and increased cardiovascular risk in Caucasians. Hum Mol Genet.

[42] Cyr Y (2020). White adipose tissue surface expression of LDLR and CD36 is associated with risk factors for type 2 diabetes in adults with obesity. Obesity (Silver Spring).

[43] Gao H (2017). CD36 is a marker of human adipocyte progenitors with pronounced adipogenic and triglyceride accumulation potential. Stem Cells.

[44] Karelis AD (2008). Metabolically healthy but obese individuals. Lancet.

[45] Denis GV, Obin MS (2013). ‘Metabolically healthy obesity’: origins and implications. Mol Aspects Med.

[46] Kim JY (2007). Obesity-associated improvements in metabolic profile through expansion of adipose tissue. J Clin Invest.

[47] Abreu-Vieira G (2015). Cidea improves the metabolic profile through expansion of adipose tissue. Nat Commun.

[48] Dalmas E (2015). Irf5 deficiency in macrophages promotes beneficial adipose tissue expansion and insulin sensitivity during obesity. Nat Med.

[49] Senol-Cosar O (2016). Tenomodulin promotes human adipocyte differentiation and beneficial visceral adipose tissue expansion. Nat Commun.

[50] Yanagida K (2018). The Gα12/13-coupled receptor LPA4 limits proper adipose tissue expansion and remodeling in diet-induced obesity. JCI Insight.

[51] Hunter AL (2021). Adipocyte NR1D1 dictates adipose tissue expansion during obesity. Elife.

[52] Santovito D (2017). Fat or fit: The big oxymoron of (metabolically) healthy obesity. Atherosclerosis.

[53] Péterfi Z (2018). Endocannabinoid and nitric oxide systems of the hypothalamic paraventricular nucleus mediate effects of NPY on energy expenditure. Mol Metab.

[54] Weir JB (1949). New methods for calculating metabolic rate with special reference to protein metabolism. J Physiol.

[55] Bruss MD (2010). Calorie restriction increases fatty acid synthesis and whole body fat oxidation rates. Am J Physiol Endocrinol Metab.

[56] Arch JR (2006). Some mathematical and technical issues in the measurement and interpretation of open-circuit indirect calorimetry in small animals. Int J Obes (Lond).

[57] Guinez C (2011). O-GlcNAcylation increases ChREBP protein content and transcriptional activity in the liver. Diabetes.

[58] Maneglier B (2004). Simultaneous measurement of kynurenine and tryptophan in human plasma and supernatants of cultured human cells by HPLC with coulometric detection. Clin Chem.

[59] Roca-Rivada A (2013). FNDC5/irisin is not only a myokine but also an adipokine. PLoS One.

[60] Tang QQ (2004). Commitment of C3H10T1/2 pluripotent stem cells to the adipocyte lineage. Proc Natl Acad Sci U S A.

[61] Hindi L (2017). Isolation, culturing, and differentiation of primary myoblasts from skeletal muscle of adult mice. Bio Protoc.

[62] Atkinson BJ (2013). Moderate GLUT4 overexpression improves insulin sensitivity and fasting triglyceridemia in high-fat diet-fed transgenic mice. Diabetes.

[63] Schindelin J (2012). Fiji: an open-source platform for biological-image analysis. Nat Methods.

[64] Reggio S (2016). Increased basement membrane components in adipose tissue during obesity: links with TGFβ and metabolic phenotypes. J Clin Endocrinol Metab.

