# Supplementary material for: Whole-body deletion of Endospanin 1 protects from obesity-associated deleterious metabolic alterations
Source: JCI Insight. 2024 May 8;9(9):e168418. doi: 10.1172/jci.insight.168418 (PMC11141941; doi:10.1172/jci.insight.168418)
Supplement: Supplemental data [file jciinsight-9-168418-s121.pdf]

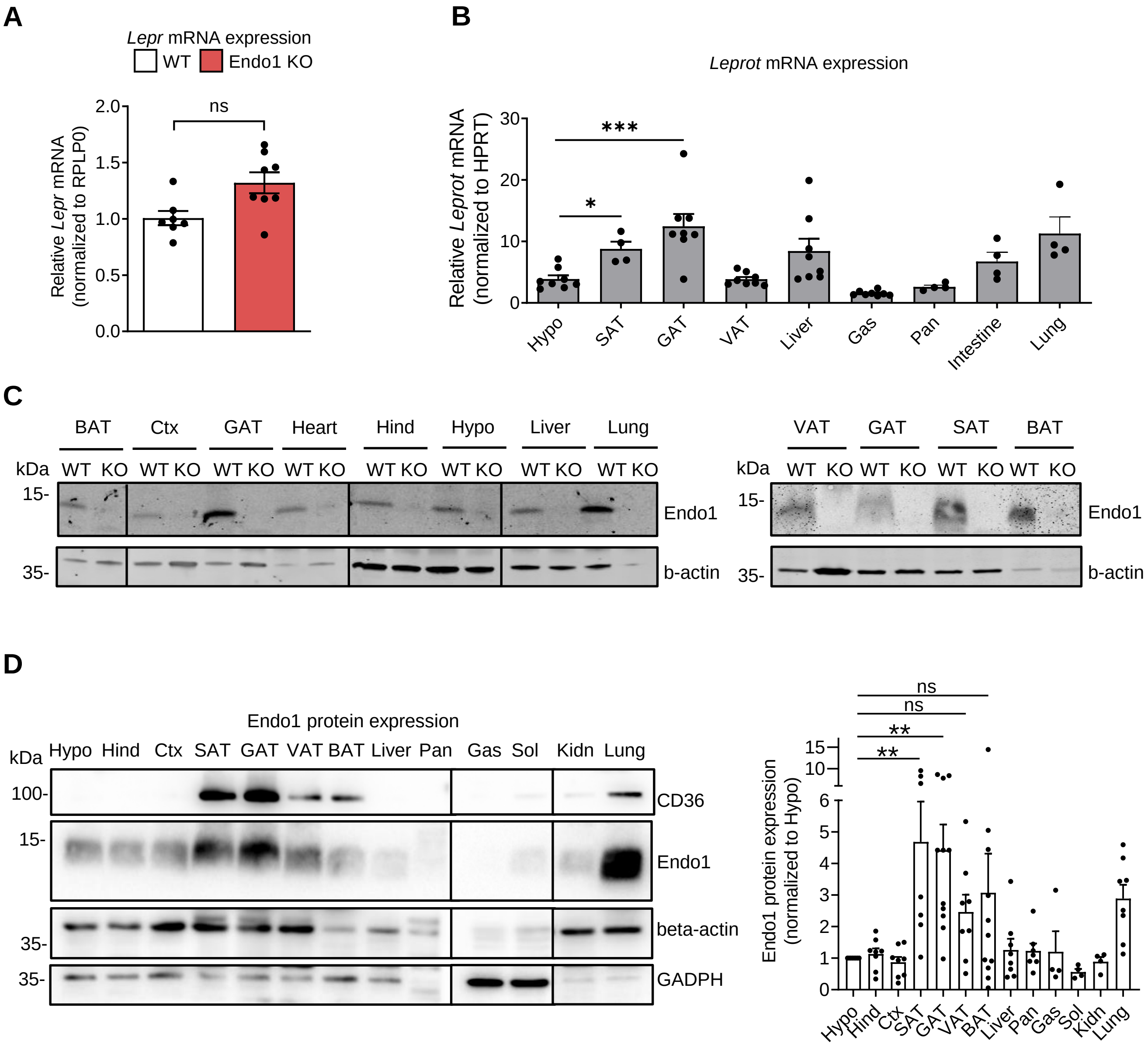

Supplemental Figure 1: **Expression of endospanin 1 and CD36 in tissues. (A)**. *Lepr* mRNA level in the hypothalamus of WT and Endo1 KO mice. Results are expressed as means ± SEM (n≥7). Two-tailed t-test. **(B)** Relative mRNA expression analyzed by RT-qPCR of *Leprot* in multiple tissues. Results are expressed as means ± SEM (n≥4). *, $p < 0.05$; and ***, $p < 0.005$ vs hypothalamus. One-way Anova followed by a two-stage linear step-up procedure of Benjamini, Krieger and Yekutieli to correct for multiple comparisons. **(C)** Endospanin 1 protein expression in WT and KO mice. Endo1 KO tissues were used as negative control. **(D)** CD36 and Endo1 protein expression in mouse tissues analyzed by immunoblot and densitometry analysis of endospanin 1 in mouse tissues. Results are expressed as means ± SEM (n≥4). **, $p < 0.01$ vs hypothalamus. One way ANOVA followed by a two-stage linear step-up procedure of Benjamini, Krieger and Yekutieli to correct for multiple comparisons. Hypothalamus (hypo), hindbrain (Hind), cortex (Ctx), adipose tissues: subcutaneous (SAT), gonadal (GAT), visceral (VAT), brown (BAT), pancreas (Pan), gastrocnemius muscle (Gas), soleus muscle (sol), kidney (Kidn). The molecular weights of protein markers are indicated (kDa).

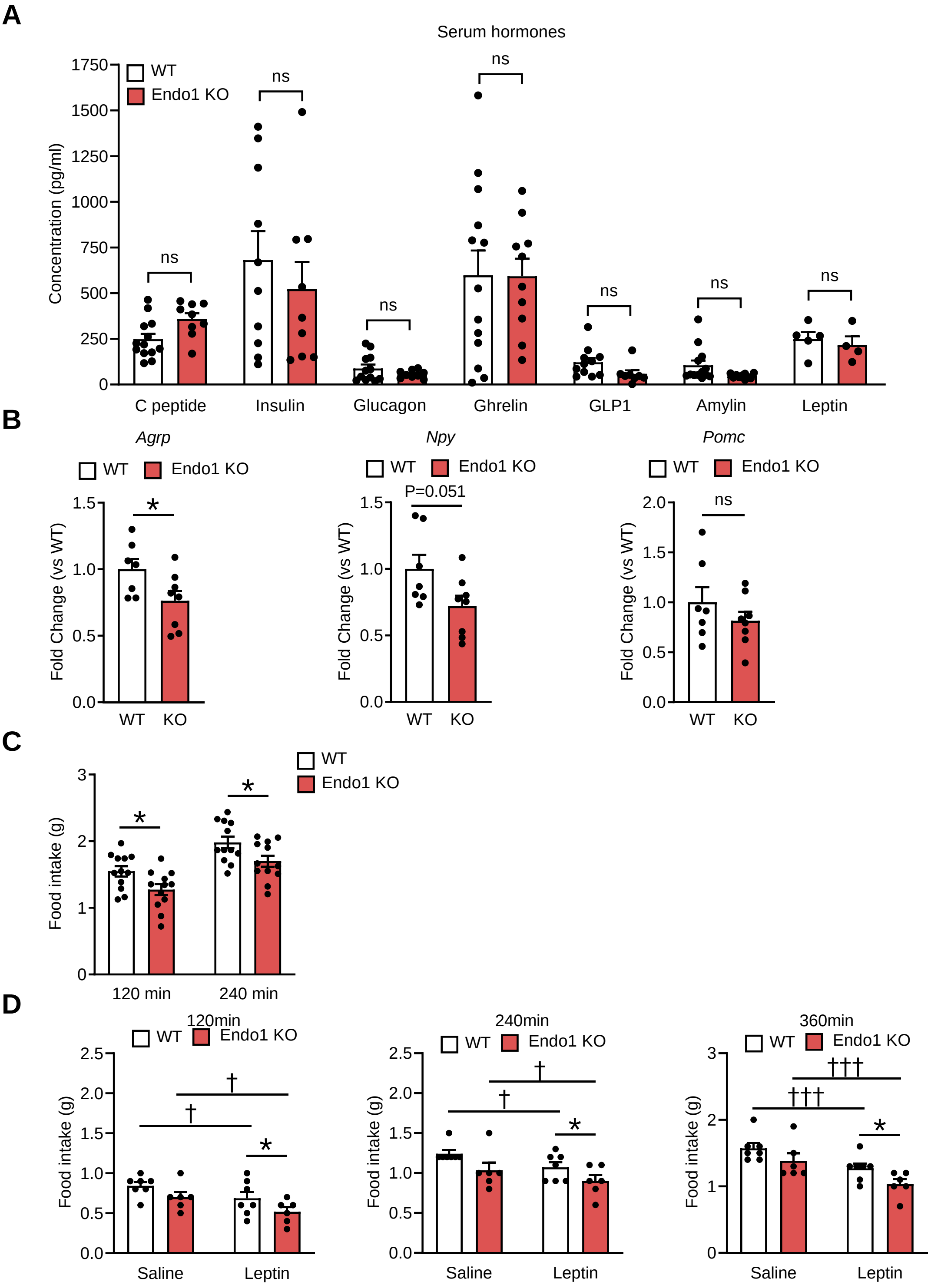

Supplemental Figure 2: Leptin action in Endo1 KO mice on chow diet. **(A)** Plasma level of C-peptide, Insulin, glucagon, ghrelin, GLP1, amylin and leptin in Endo1 KO and WT mice. Results are expressed as means ± SEM (n≥4). Two-tailed t-test. **(B)** 12h-fasting induced mRNA levels of *Agrp*, *Npy* and *Pomc* in the hypothalamic arcuate nucleus punches. Results are expressed as means ± SEM (n≥7). *, $p < 0.01$ vs WT. Two-tailed t-test. **(C)** Food intake after 12 hour-fasting. Results are expressed as means ± SEM (n=12). *, $p < 0.01$ vs WT. One way ANOVA with Bonferroni correction. **(D)** Leptin-induced inhibition of food intake after Fasting-Refeeding experiment for 120min, 240min and 360min of refeeding. Results are expressed as means ± SEM (n≥6). *, $p < 0.05$ vs WT; †, $p < 0.05$, and †††, $p < 0.005$ vs saline. Anova followed by Šídák's multiple comparisons test.

A B

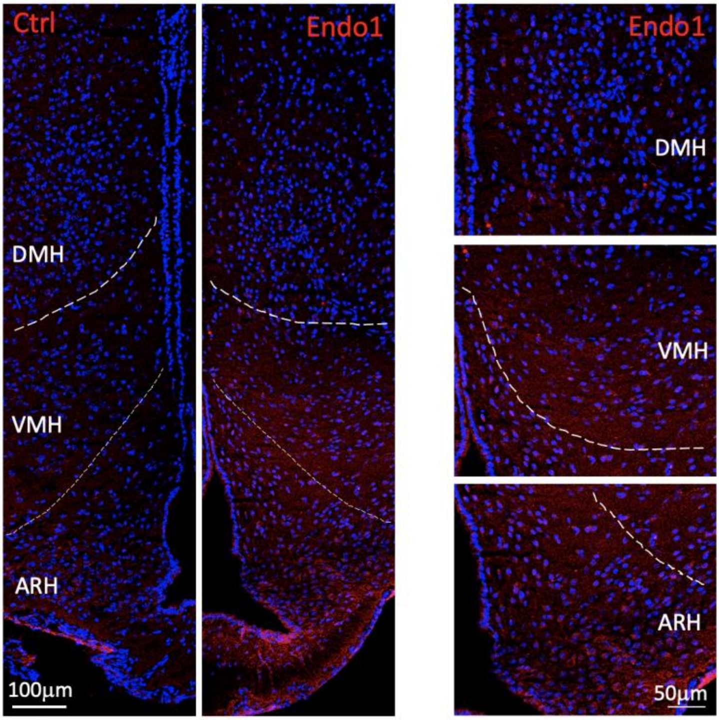

Supplemental Figure 3: Immunostaining for endospanin 1 in the mouse hypothalamus. A. Endospanin 1 is mainly detected in the the arcuate nucleus (ARH) and in few cells of the ventromedial hypothalamic nucleus (VMH) and even fewer in the dorsomedial nucleus of the hypothalamus (DMH). Ctrl, control without primary antibody. **B.** higher magnification of the ARH, VMH and DMH regions of the medio-basal hypothalamus after immunostaining for endospanin 1. Nuclei (blue) are conterstained with fluorescent dapi dye.

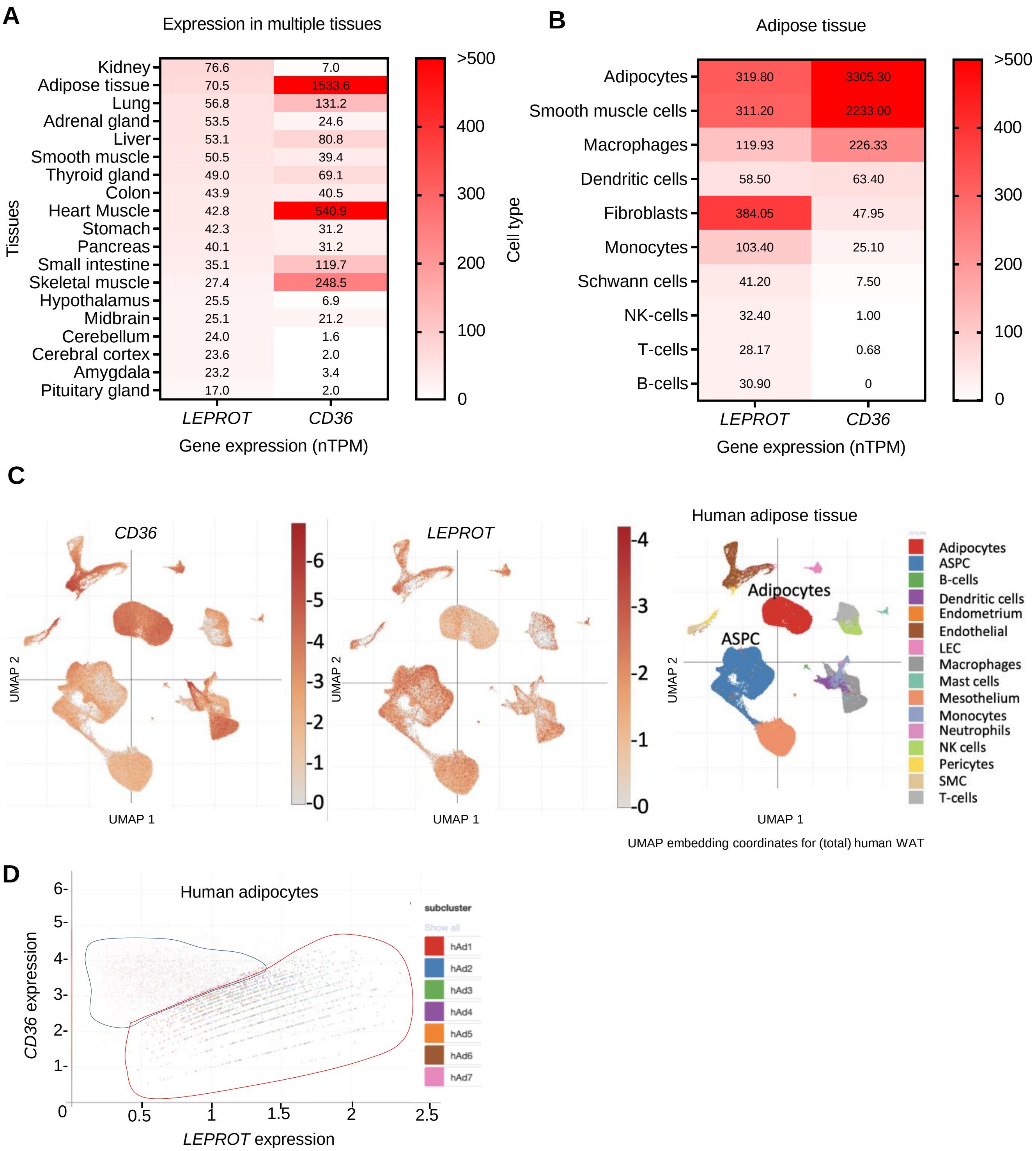

Supplemental Figure 4: *LEPROT*, and *CD36* mRNA expression in human tissues and single-cell analysis. (A) mRNA expression in human tissues. **(B)** mRNA expression from single-cell analysis of human adipose tissue. Data from (A) and (B) were obtained from the Human Protein Atlas (20). nTPM: Normalized transcripts per million. **(C)** mRNA expression patterns of *CD36* and *LEPROT* gene in human adipose tissue from single cell RNAseq of (21). (**D**) Correlation between *CD36* and *LEPROT* in human adipocytes from single cell analysis of (21). Different subclusters of adipocytes (hAd1-7) are depicted. A positive correlation is seen for a subset of adipocytes highlighted in the red frame.

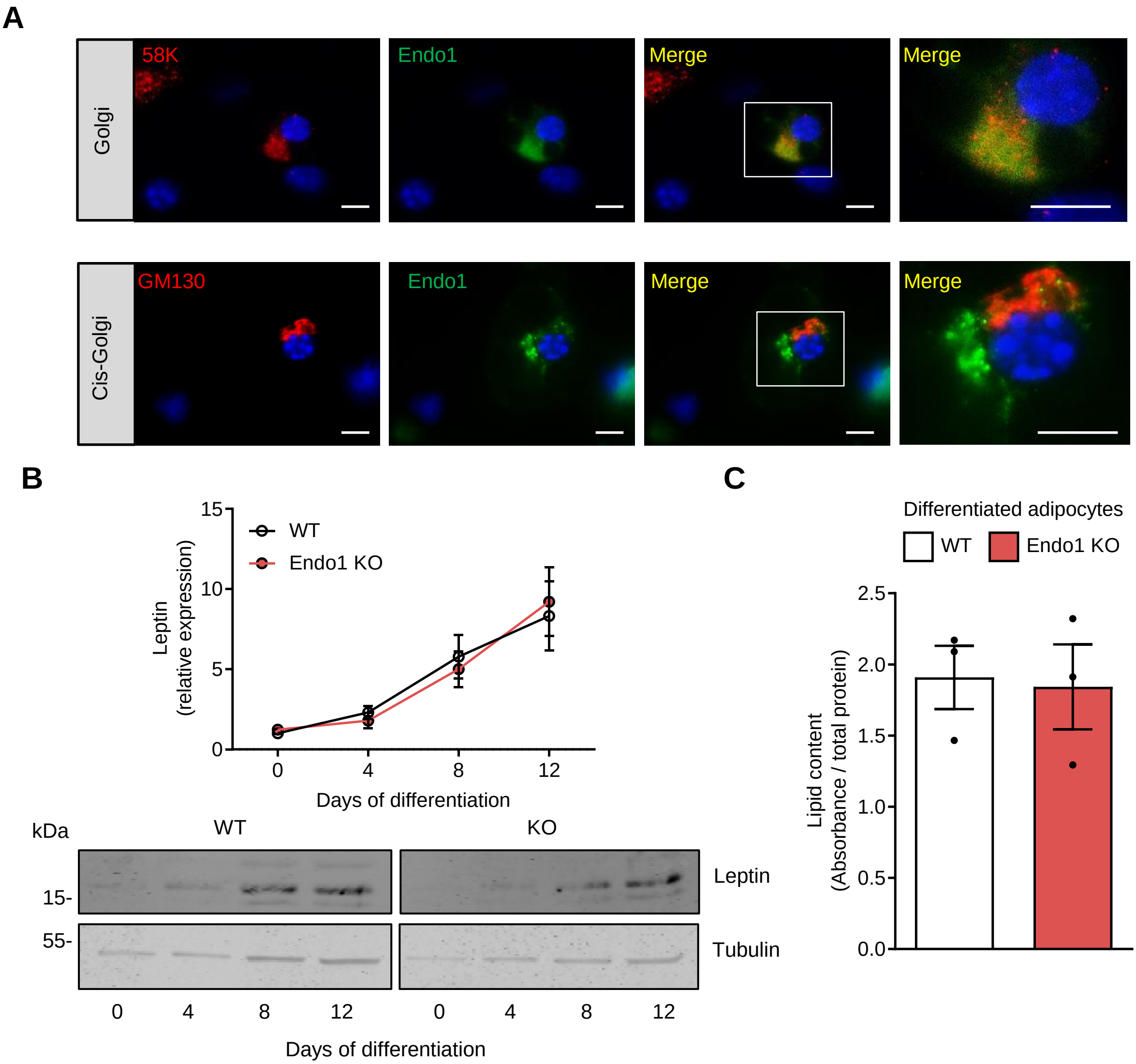

Supplemental Figure 5: **Endo1 expression does not affect adipocyte differentiation**. (**A**) Confocal immunofluorescence images of the co-localisation between endospanin 1 (rabbit anti-Endo1) and the Golgi markers 58K (total Golgi, mouse anti-58K) or GM130 (cis-Golgi, mouse anti-GM130) in differentiated white adipocytes. Nuclei (blue) are conterstained with fluorescent dapi dye. Representative images of two independent experiments. Scale is 20 µm. (**B**) Leptin protein level during adipocyte differentiation in the endo1 KO mice. Results are expressed as means ± SEM of six independent experiments (n=6); Two-way ANOVA with Bonferroni correction. The molecular weights of protein markers are indicated (kDa). (**C**) Lipid content quantification by Oil Red O staining. Results are expressed as mean ± SEM (n=3). Two-tailed t test.

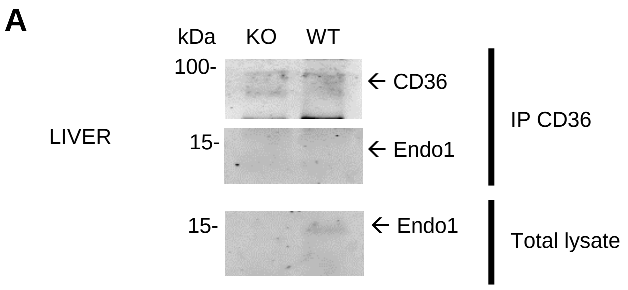

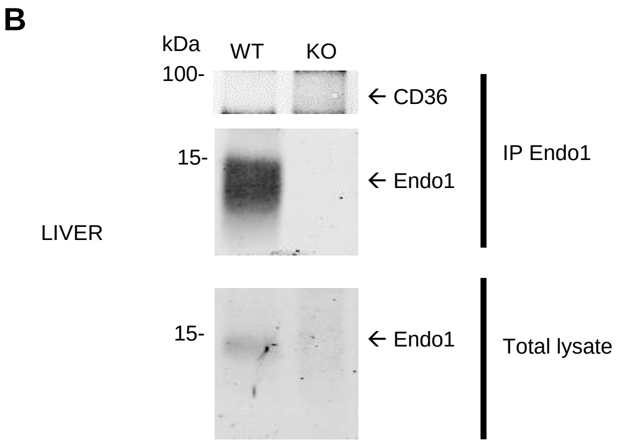

Supplemental Figure 6: Detection of endogenous endospanin 1 and CD36 after immunoprecipitation with CD36 (**A**) or endospanin 1 (**B**) antibodies from liver lysates of wild type and KO mice. Endo1 KO liver was used as negative controls. The molecular weights of protein markers are indicated (kDa). Representative blots of three independent experiments.

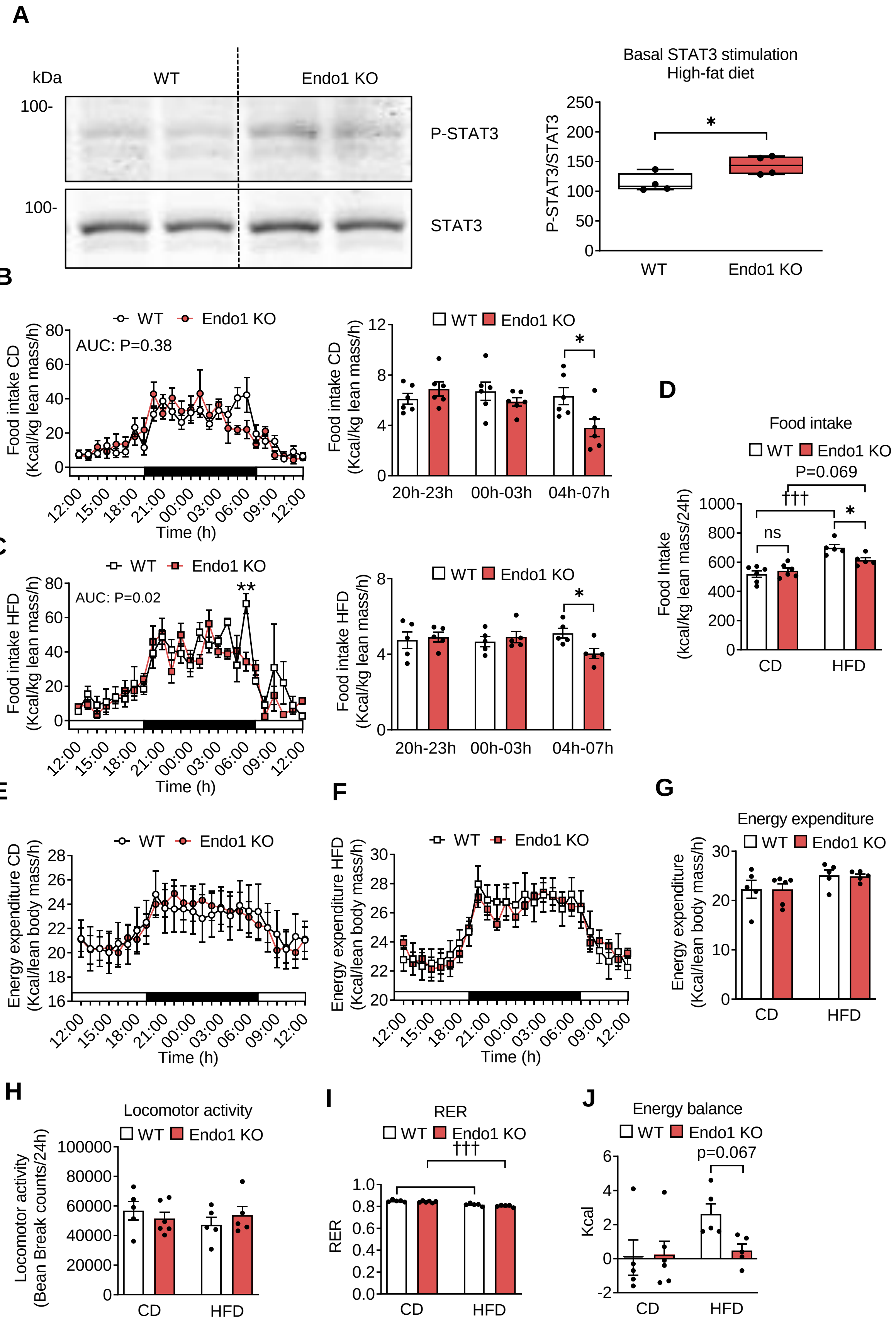
A
kDa
WT
Endo1 KO
100-
P-STAT3
100-
STAT3
Basal STAT3 stimulation
High-fat diet
P-STAT3/STAT3
*
WT
Endo1 KO
B
WT
Endo1 KO
AUC: P=0.38
Food intake CD
(Kcal/kg lean mass/h)
Time (h)
20h-23h
00h-03h
04h-07h
C
AUC: P=0.02
**
Food intake HFD
(Kcal/kg lean mass/h)
D
Food intake
P=0.069
†††
ns
Food Intake
(Kcal/kg lean mass/24h)
CD
HFD
E
Energy expenditure CD
(Kcal/lean body mass/h)
F
Energy expenditure HFD
(Kcal/lean body mass/h)
G
Energy expenditure
Energy expenditure
(Kcal/lean body mass/h)
H
Locomotor activity
Locomotor activity
(Bean Break counts/24h)
I
RER
†††
J
Energy balance
p=0.067
Kcal

Supplemental Figure 7: Leptin-related metabolic effects. (A) STAT3 phosphorylation level in the hypothalamus of Endo1 KO vs WT mice after 4-weeks HFD. Results are expressed as means ± SEM (n=4). *, $p < 0.05$ vs WT. Two-tailed t-test. **(B-C)** Calories consumed per hour followed in metabolic cages, throughout 24 hrs, on CD (B) or HFD (C), with a focus on food intake during the night time (right panel). Results are expressed as means ± SEM (n≥5). *, $p < 0.05$; and **, $p < 0.01$ vs WT. Two-way ANOVA (left panels), one way ANOVA (right panels) with Bonferroni correction. **(D)** Total calories consumed per day. Results are expressed as means ± SEM (n≥5). *, $p < 0.05$ vs WT; †††, $p<0.005$ vs CD. One way ANOVA with Bonferroni correction. **(E-F)** Energy expenditure throughout 24 hrs on CD (left panel) or HFD (right panel)**.** Results are expressed as means ± SEM (n≥5). Two-way ANOVA with Bonferroni correction. **(G)** Mean energy expenditure per hour**.** Results are expressed as means ± SEM (n≥5). Two-way ANOVA with Bonferroni correction. **(H)** Average locomotor activity per 24 hrs. Results are expressed as means ± SEM (n≥5). One way ANOVA with Bonferroni correction. **(I)** Average respiratory exchange ratio (RER). Results are expressed as means ± SEM (n≥5). †††, $p<0.001$ vs CD. One way ANOVA with Bonferroni correction. **(J)** Energy balance. Results are expressed as means ± SEM (n≥5). One-way ANOVA with Bonferroni correction.

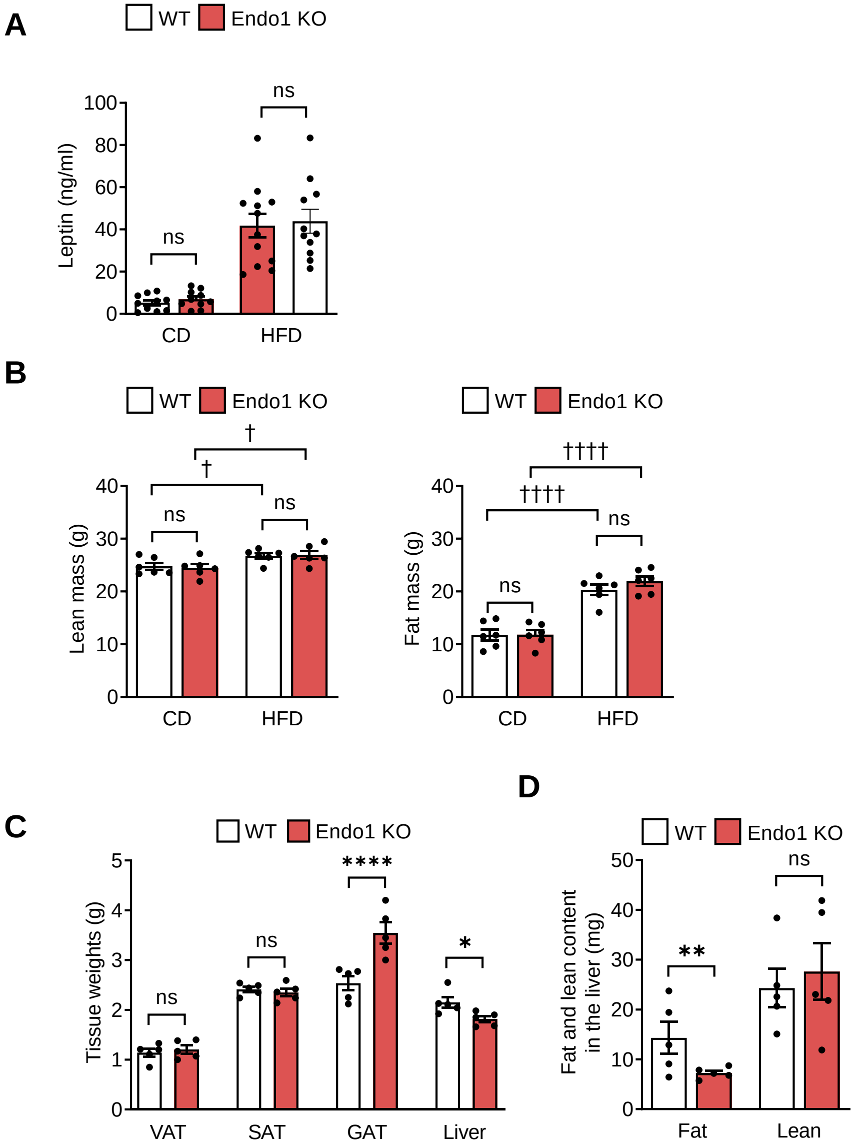

Supplemental Figure 8: Leptinemia and fat content. (A) Leptinemia of WT and Endo1 KO mice fed a HFD or CD. Results are expressed as means ± SEM (n≥10). One-way ANOVA with Bonferroni correction. **(B)** Absolute values in g of Figure 3B. Results are expressed as means ± SEM (n=6). †, p<0.05; and ††††, p<0.001 vs CD. One-way ANOVA with Bonferroni correction. **(C)** absolute values in g of Figure 3C. Results are expressed as means ± SEM (n=5). *, p < 0.05; ****; and p < 0.001 vs WT. One-way ANOVA with Bonferroni correction. **(D)** absolute values in mg of Figure 3D. Results are expressed as means ± SEM (n=5). **, p < 0.01 vs WT. One-way ANOVA with Bonferroni correction.

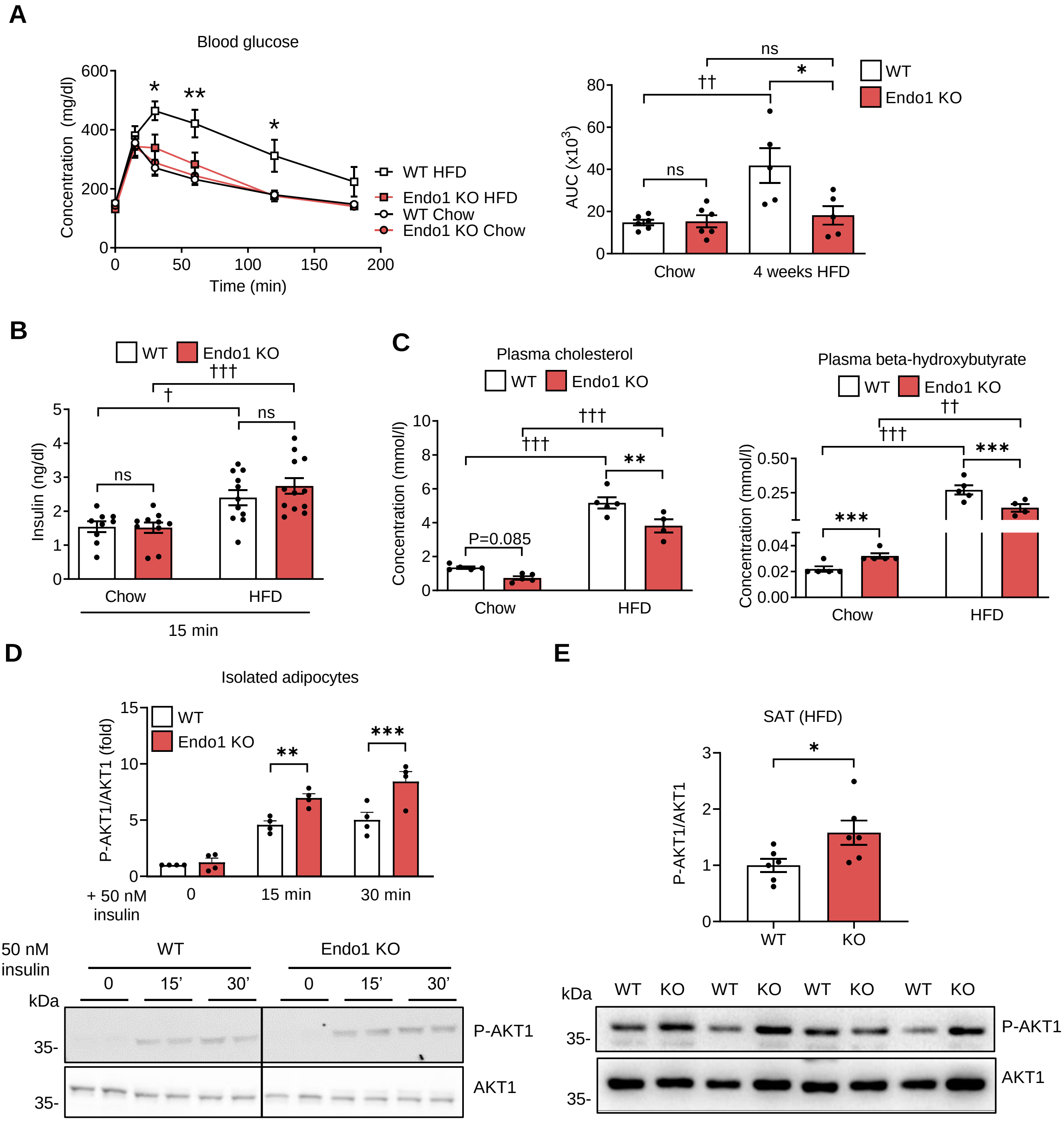
A
Blood glucose
Concentration (mg/dl)
Time (min)
WT HFD
Endo1 KO HFD
WT Chow
Endo1 KO Chow
AUC (x10^3)
WT
Endo1 KO
Chow
4 weeks HFD
B
Insulin (ng/dl)
Chow
HFD
15 min
C
Plasma cholesterol
Concentration (mmol/l)
P=0.085
Plasma beta-hydroxybutyrate
D
Isolated adipocytes
P-AKT1/AKT1 (fold)
+ 50 nM insulin
0
15 min
30 min
50 nM insulin
kDa
P-AKT1
AKT1
E
SAT (HFD)
P-AKT1/AKT1
WT
KO

Supplemental Figure 9: Glucose homeostasis. (A) Intraperitoneal glucose tolerance test (ip-GTT) in Endo1 KO and WT mice after a 4-week high-fat diet. Results are expressed as means ± SEM ($n \geq 5$). *, $p < 0.05$; and **, $p < 0.01$ vs WT. Two-way ANOVA with Bonferroni correction (left panel); and area under the curve of ip-GTT curves (right panel). *, $p < 0.05$ vs WT. ††, $p < 0.01$ vs CD. One-way ANOVA with Bonferroni correction. **(B**) Plasma insulin at 15min following ip-injection of glucose (2 g/kg) in Endo1 KO and WT mice on chow diet or after 10-week of HFD. Results are expressed as means ± SEM ($n \geq 9$). †, $p<0.05$, and †††, $p<0.005$ vs CD. Two-way ANOVA with Turkey's multiple comparison test. **(C)** Plasma level of Cholesterol and b-hydroxybutyrate. Results are expressed as means ± SEM ($n \geq 4$). **, $p < 0.01$; and ***, $p < 0.001$ vs WT; ††, $p < 0.01$, and †††, $p < 0.001$ vs control. One-way ANOVA with Bonferroni correction. **(D)** Phosphorylation of AKT in isolated adipocytes stimulated with 50nM insulin for 15 or 30 minutes. Results are expressed as means ± SEM (n=4). **, $p < 0.01$; and ***, $p < 0.001$ vs WT. One way ANOVA with Bonferroni correction. **(E)** Phosphorylation of AKT in the subcutaneaous adipose tissue of HFD mice. Results are expressed as means ± SEM (n=6). *, $p< 0.05$ vs WT. Unpaired T-test.

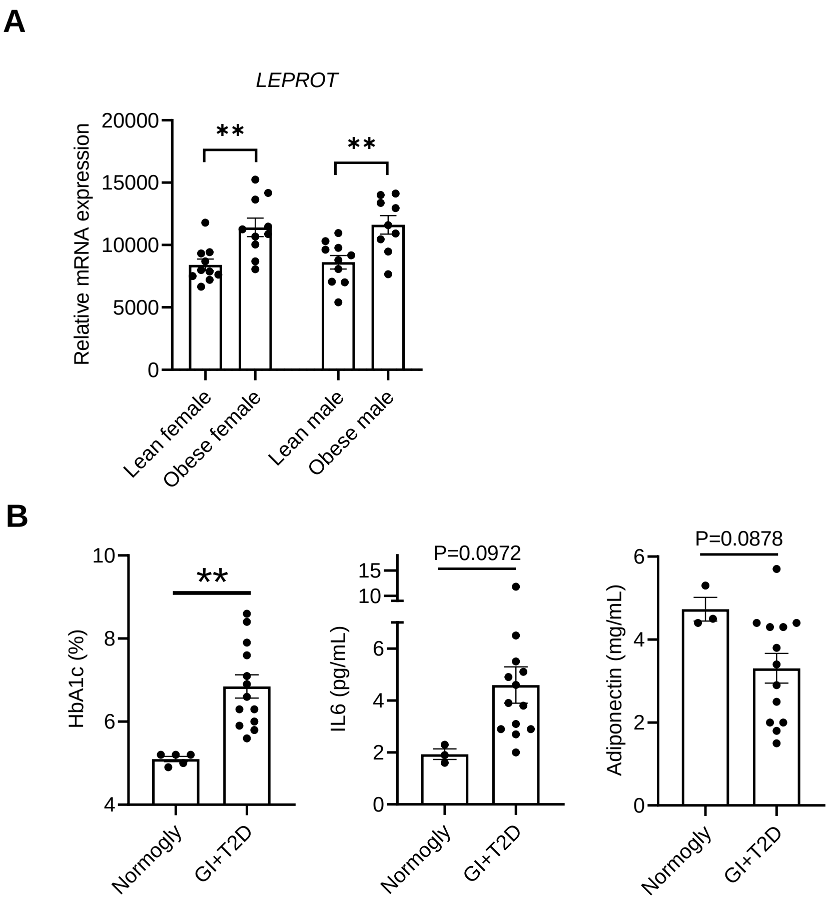

Supplemental Figure 10: Leprot expression in humans. (A) *LEPROT* mRNA expression in mature adipocytes of subcutaneous adipose tissue from obese individuals from Gene Expression Omnibus (GEO) GSE2508 (GPL92) dataset. Results are expressed as means ± SEM (n≥9). **, p < 0.01 vs lean. One-way ANOVA with Turkey's post hoc test. **(B)** Plasma levels of HbA1c, IL6 and adiponectin in obese patients with impaired glucose tolerance or T2D ("GI+T2D") versus normoglycaemic obese patients ("normogly"). Results are expressed as means ± SEM (n≥3). **, p < 0.01 vs normogly. Two-tailed t-test.
