# Supplementary material for: Whole-body deletion of Endospanin 1 protects from obesity-associated deleterious metabolic alterations
Source: JCI Insight. 2024 May 8;9(9):e168418. doi: 10.1172/jci.insight.168418 (PMC11141941; doi:10.1172/jci.insight.168418)
Supplement: Unedited blot and gel images [file jciinsight-9-168418-s122.pptx]

## Slide 1
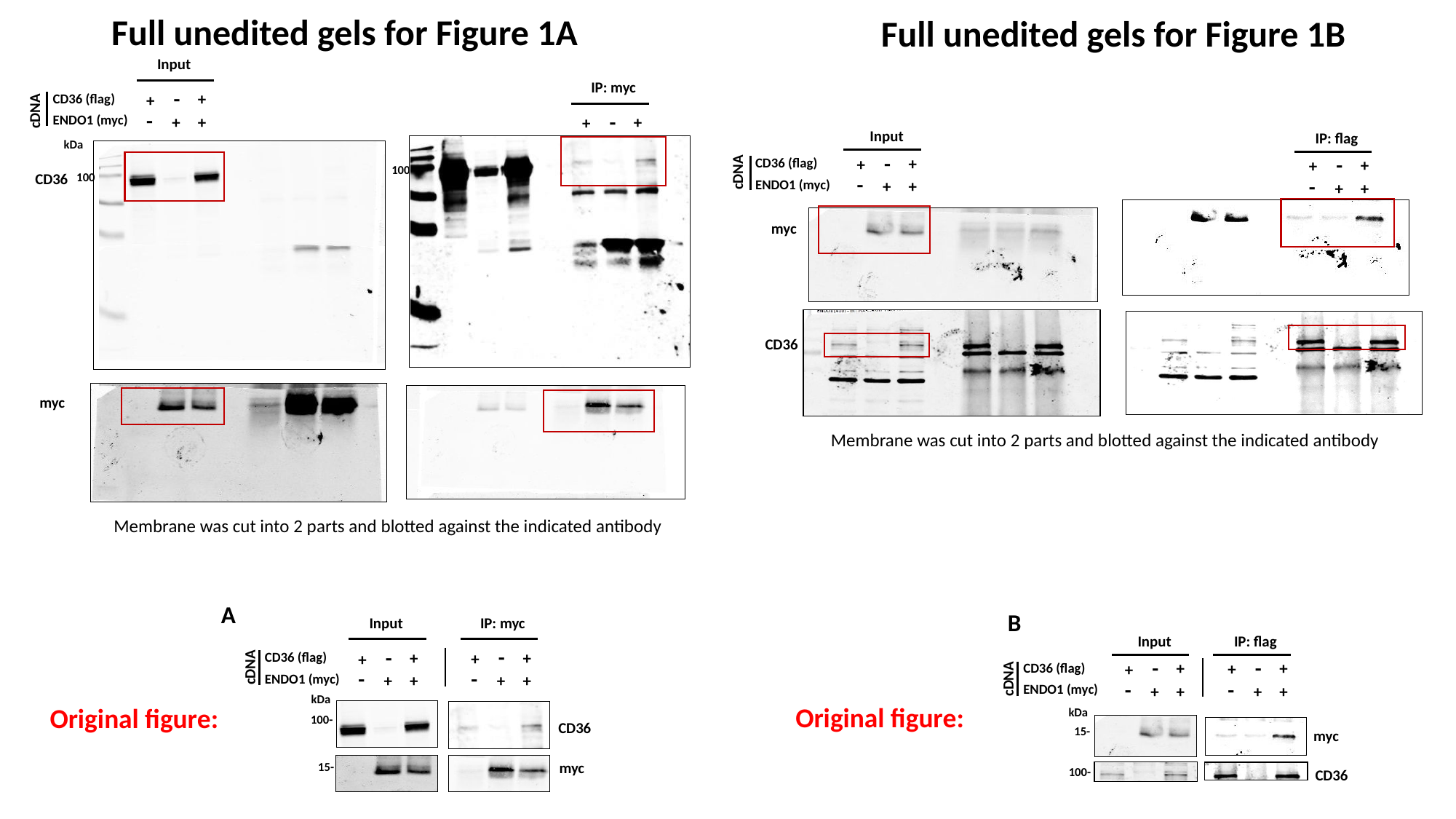

Full unedited gels for Figure 1A
Full unedited gels for Figure 1B
Input
-
+
+
CD36 (flag)
cDNA
-
ENDO1 (myc)
+
+
kDa
IP: myc
-
+
+
-
+
+
Input
IP: flag
-
-
+
+
CD36 (flag)
+
+
100
cDNA
CD36
100
-
-
ENDO1 (myc)
+
+
+
+
myc
CD36
myc
Membrane was cut into 2 parts and blotted against the indicated antibody
Membrane was cut into 2 parts and blotted against the indicated antibody
A
Input
IP: myc
-
-
+
+
+
+
CD36 (flag)
cDNA
-
-
ENDO1 (myc)
+
+
+
+
kDa
100-
CD36
myc
15-
B
Input
IP: flag
-
+
+
-
+
+
-
+
+
CD36 (flag)
cDNA
-
ENDO1 (myc)
+
+
kDa
15-
myc
100-
CD36
Original figure:
Original figure:

## Slide 2
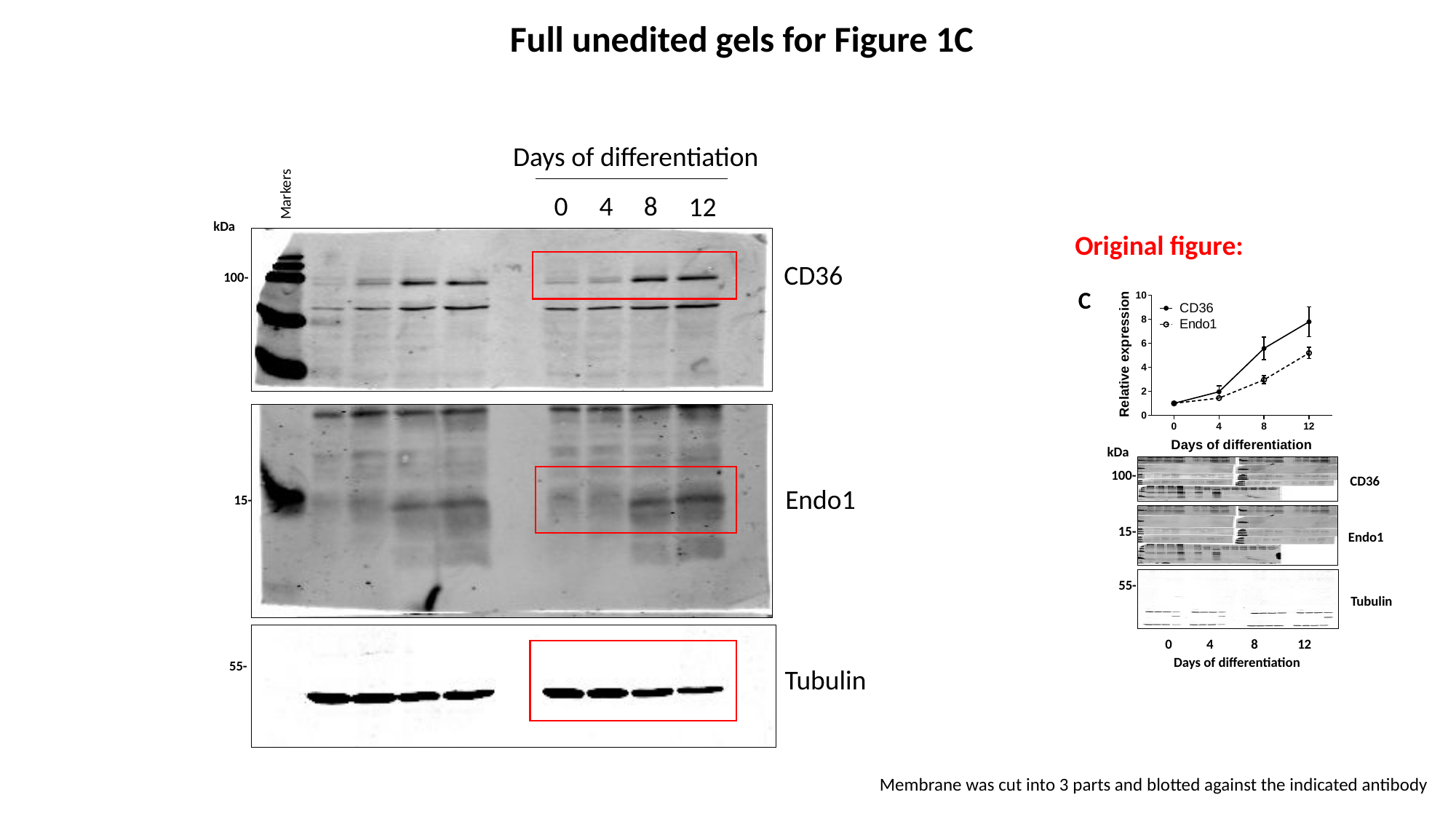

Full unedited gels for Figure 1C
Days of differentiation
Markers
0
4
8
12
kDa
Original figure:
CD36
100-
kDa
100-
CD36
15-
Endo1
55-
Tubulin
0
4
8
12
Days of differentiation
C
Endo1
15-
55-
Tubulin
Membrane was cut into 3 parts and blotted against the indicated antibody

## Slide 3
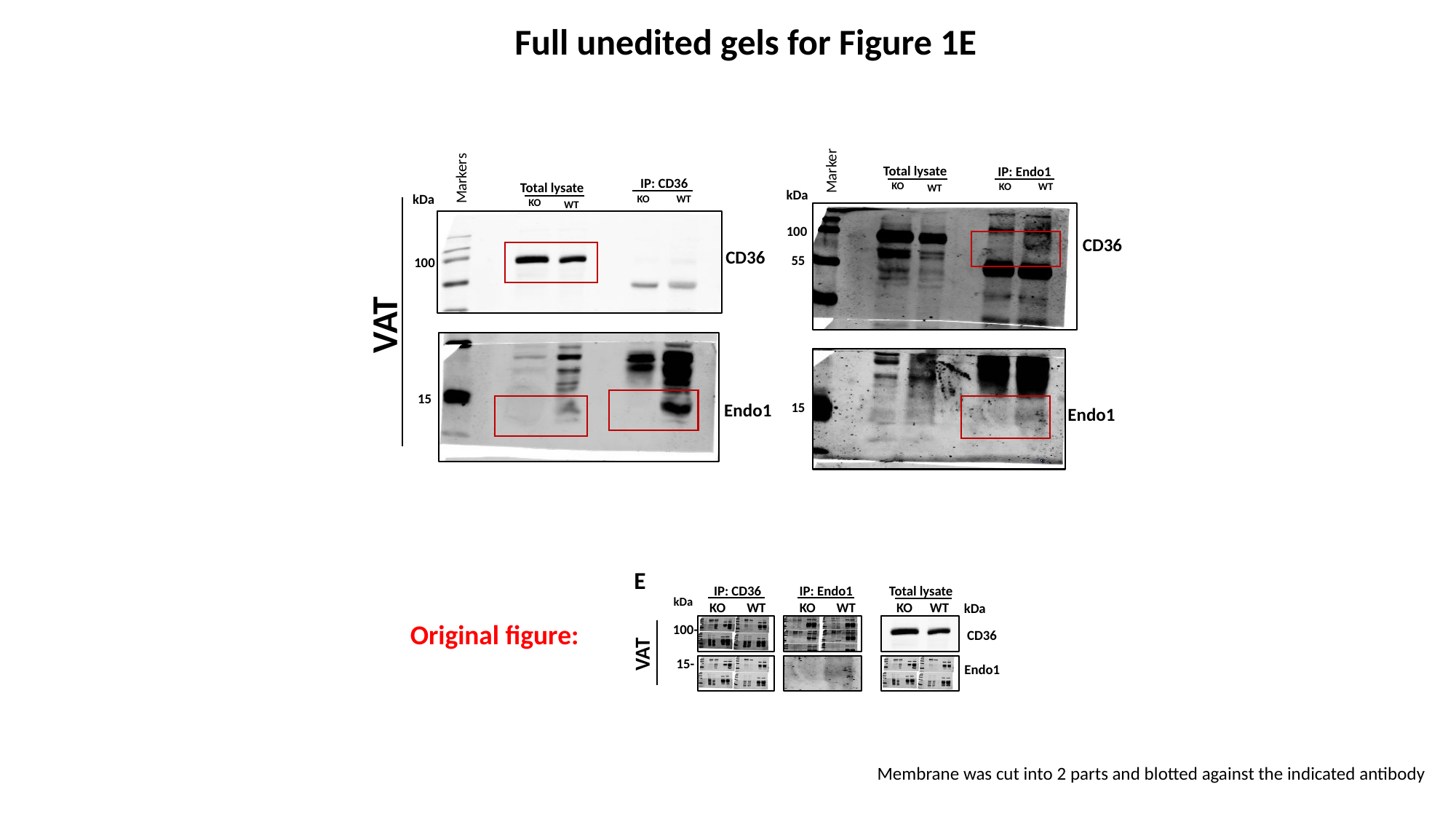

Full unedited gels for Figure 1E
Marker
Total lysate
IP: Endo1
Markers
IP: CD36
Total lysate
KO
KO
WT
WT
kDa
kDa
KO
WT
KO
WT
100
CD36
CD36
55
100
VAT
15
Endo1
15
Endo1
E
IP: CD36
IP: Endo1
Total lysate
kDa
KO
KO
WT
KO
WT
WT
kDa
100-
CD36
VAT
15-
Endo1
Original figure:
Membrane was cut into 2 parts and blotted against the indicated antibody

## Slide 4
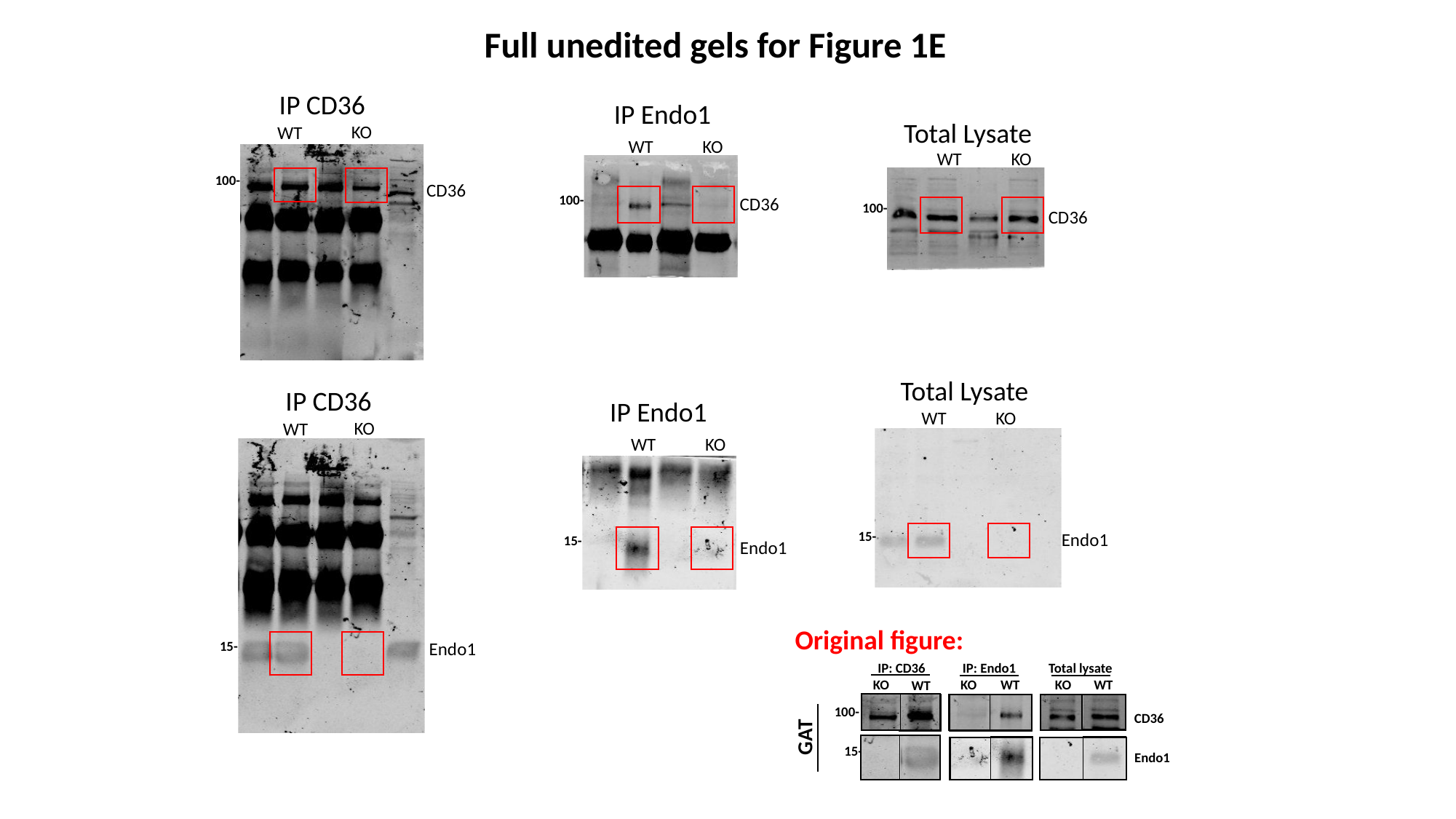

Full unedited gels for Figure 1E
IP CD36
KO
WT
100-
CD36
IP Endo1
KO
WT
100-
CD36
Total Lysate
KO
WT
100-
CD36
Total Lysate
KO
WT
15-
Endo1
IP CD36
KO
WT
Endo1
15-
IP Endo1
KO
WT
15-
Endo1
Original figure:
Total lysate
IP: CD36
IP: Endo1
KO
KO
WT
KO
WT
WT
100-
CD36
GAT
15-
Endo1

## Slide 5
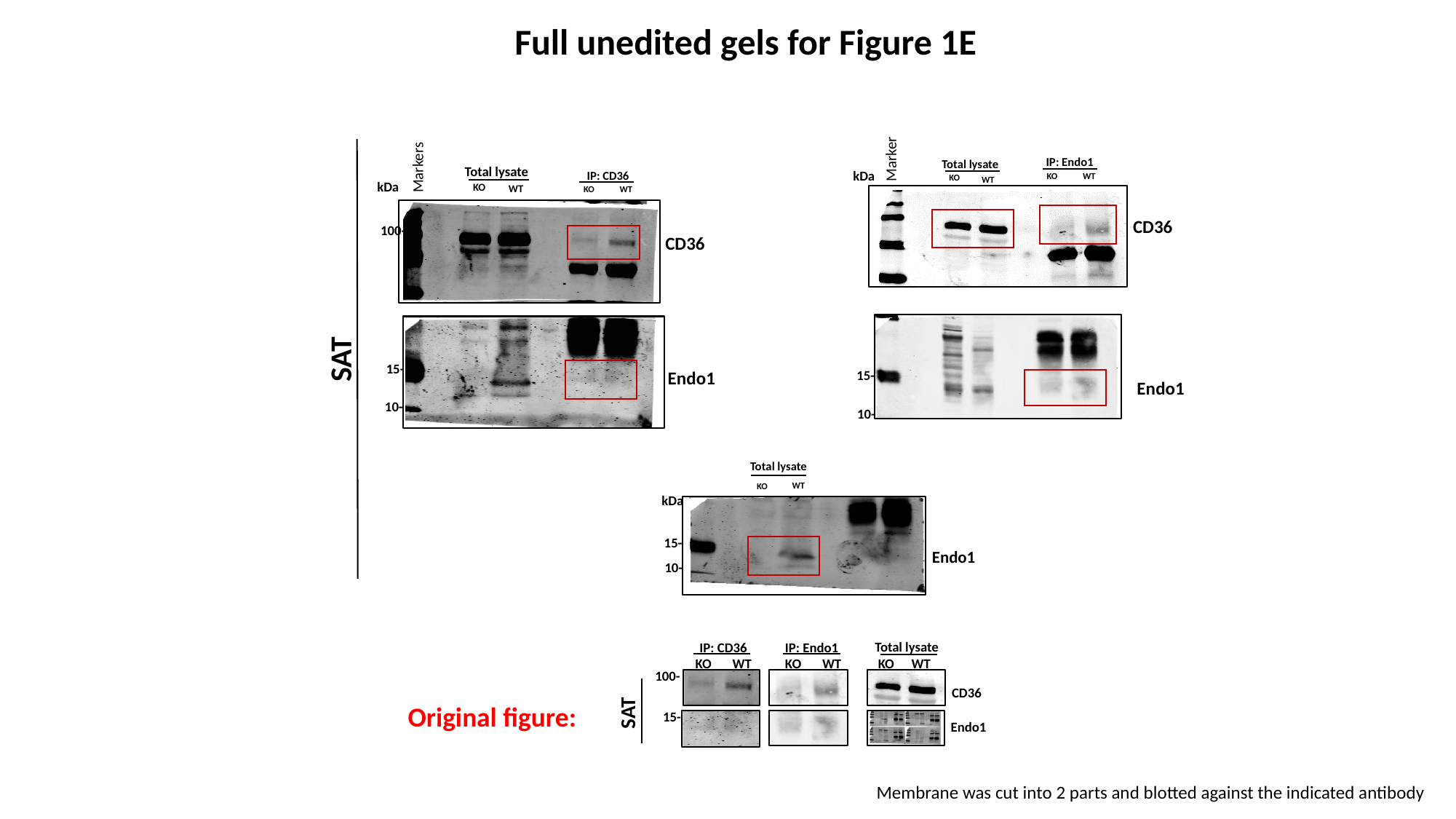

Full unedited gels for Figure 1E
Marker
IP: Endo1
Total lysate
Markers
Total lysate
kDa
IP: CD36
KO
WT
KO
WT
kDa
KO
WT
KO
WT
CD36
100-
CD36
SAT
15-
Endo1
15-
Endo1
10-
10-
Total lysate
WT
KO
kDa
15-
Endo1
10-
Total lysate
IP: CD36
IP: Endo1
KO
KO
WT
KO
WT
WT
100-
CD36
SAT
15-
Endo1
Original figure:
Membrane was cut into 2 parts and blotted against the indicated antibody

## Slide 6
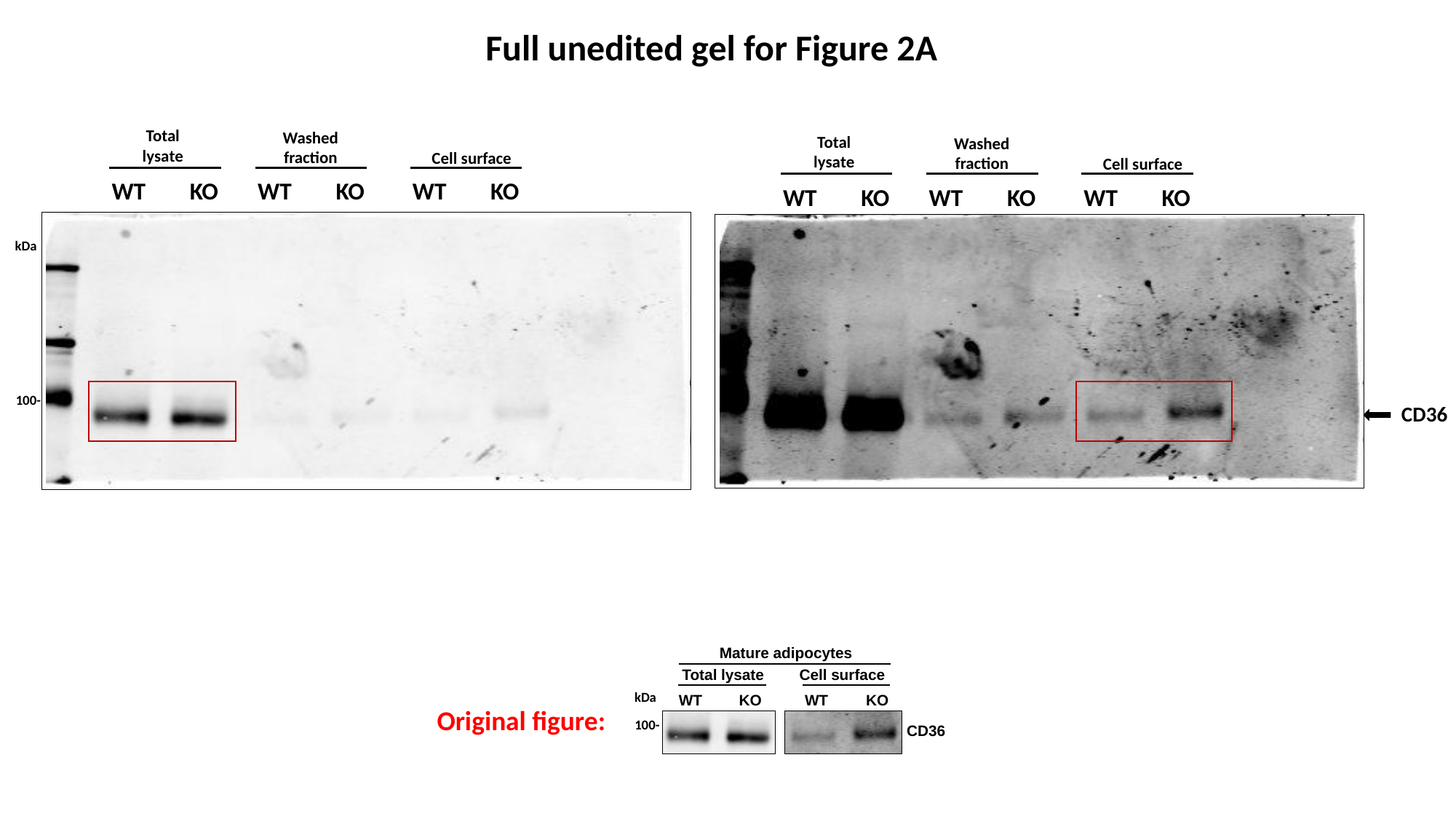

Full unedited gel for Figure 2A
Total
lysate
Washed
fraction
Total
lysate
Washed
fraction
Cell surface
Cell surface
WT
KO
WT
KO
WT
KO
WT
KO
WT
KO
WT
KO
kDa
100
100-
CD36
Mature adipocytes
Total lysate
Cell surface
kDa
WT
KO
WT
KO
100-
CD36
Original figure:

## Slide 7
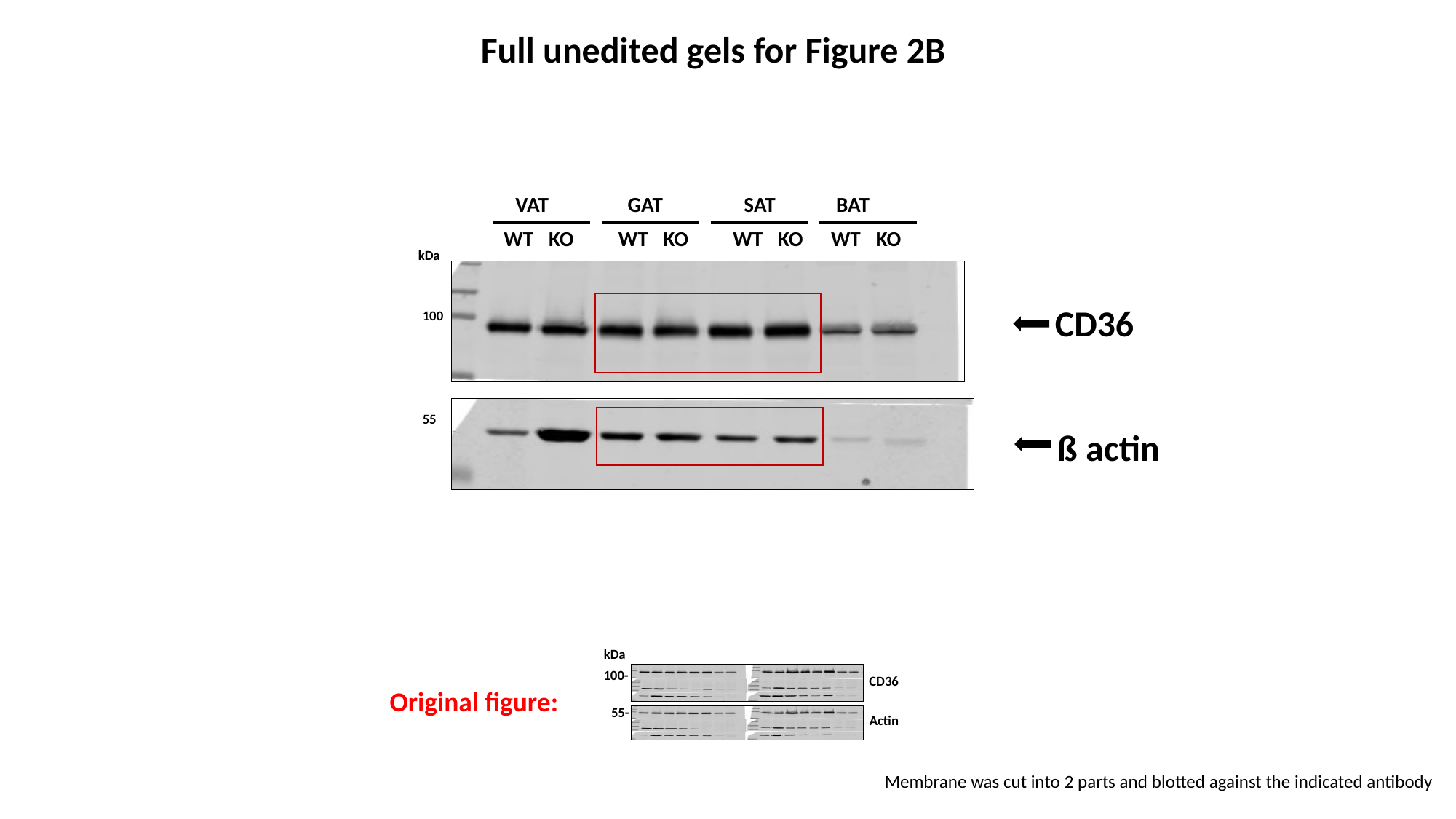

Full unedited gels for Figure 2B
VAT
GAT
SAT
BAT
WT
KO
WT
KO
WT
KO
WT
KO
kDa
CD36
100
55
ß actin
kDa
100-
55-
CD36
Actin
Original figure:
Membrane was cut into 2 parts and blotted against the indicated antibody

## Slide 8
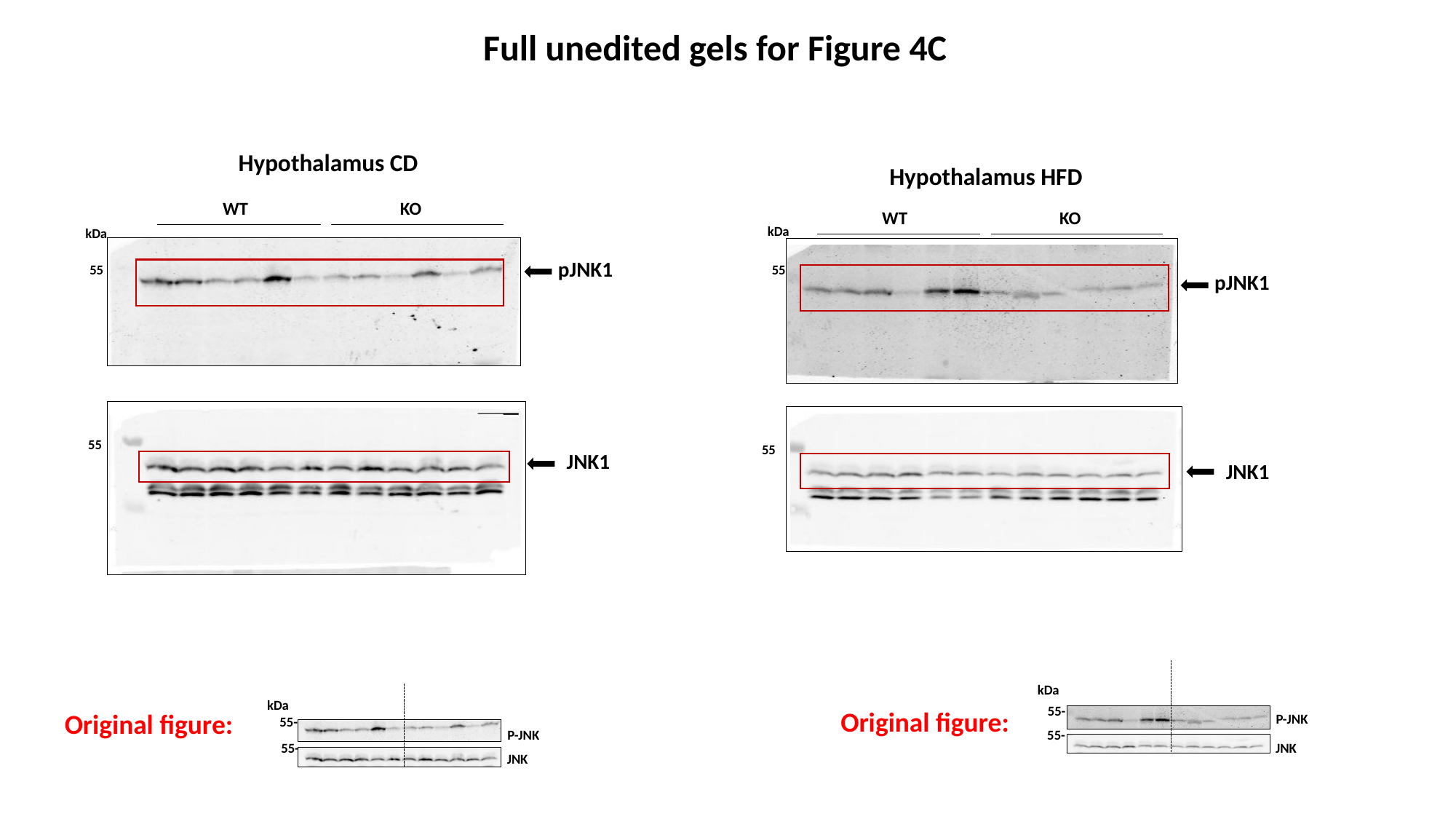

Full unedited gels for Figure 4C
Hypothalamus CD
Hypothalamus HFD
WT
KO
WT
KO
kDa
kDa
pJNK1
55
55
pJNK1
55
55
JNK1
JNK1
kDa
55-
P-JNK
55-
JNK
kDa
55-
P-JNK
55-
JNK
Original figure:
Original figure:

## Slide 9
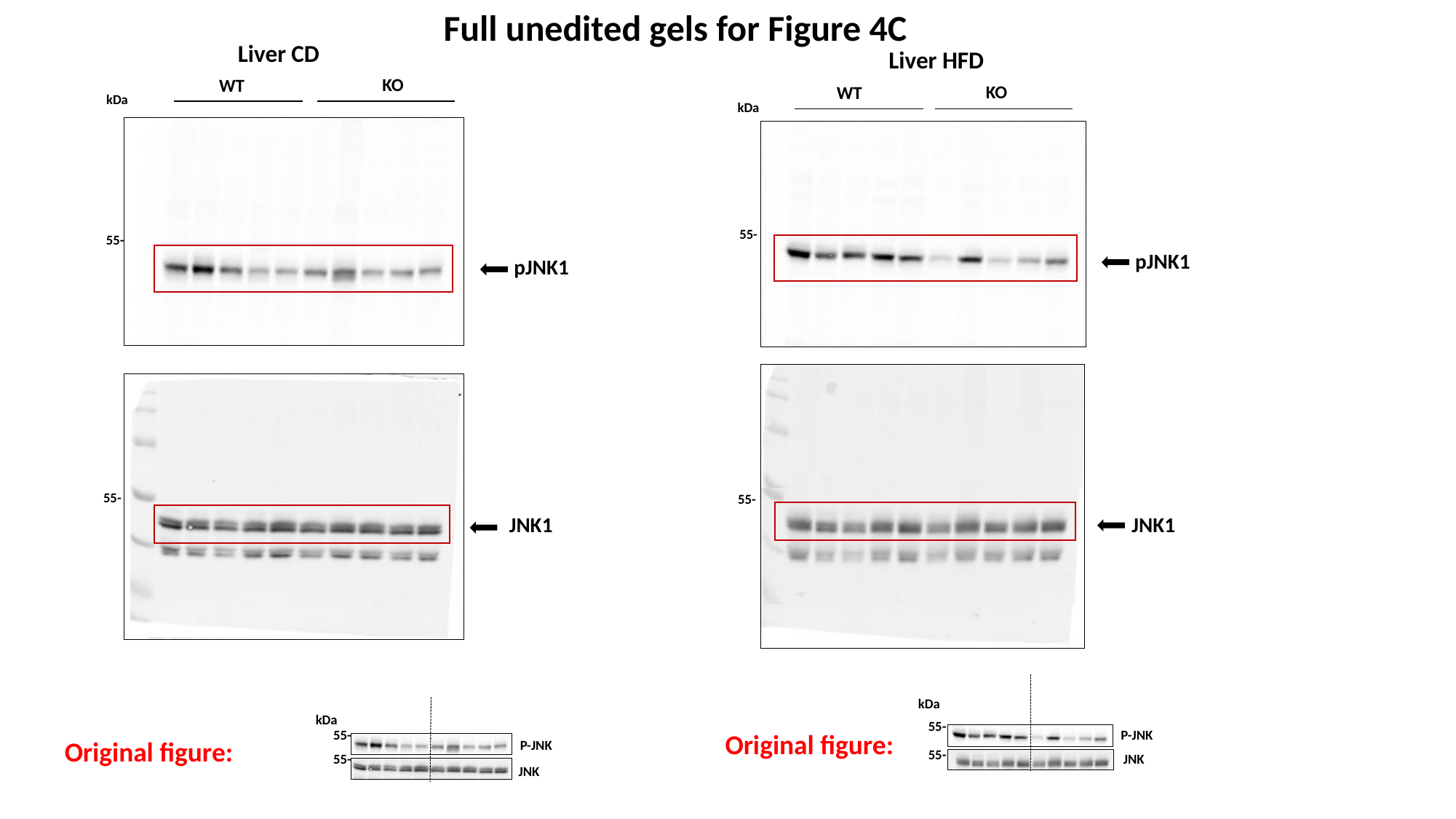

Full unedited gels for Figure 4C
Liver CD
Liver HFD
KO
WT
KO
WT
kDa
kDa
55-
55-
pJNK1
pJNK1
55-
55-
JNK1
JNK1
kDa
55-
P-JNK
55-
JNK
kDa
55-
P-JNK
55-
JNK
Original figure:
Original figure:

## Slide 10
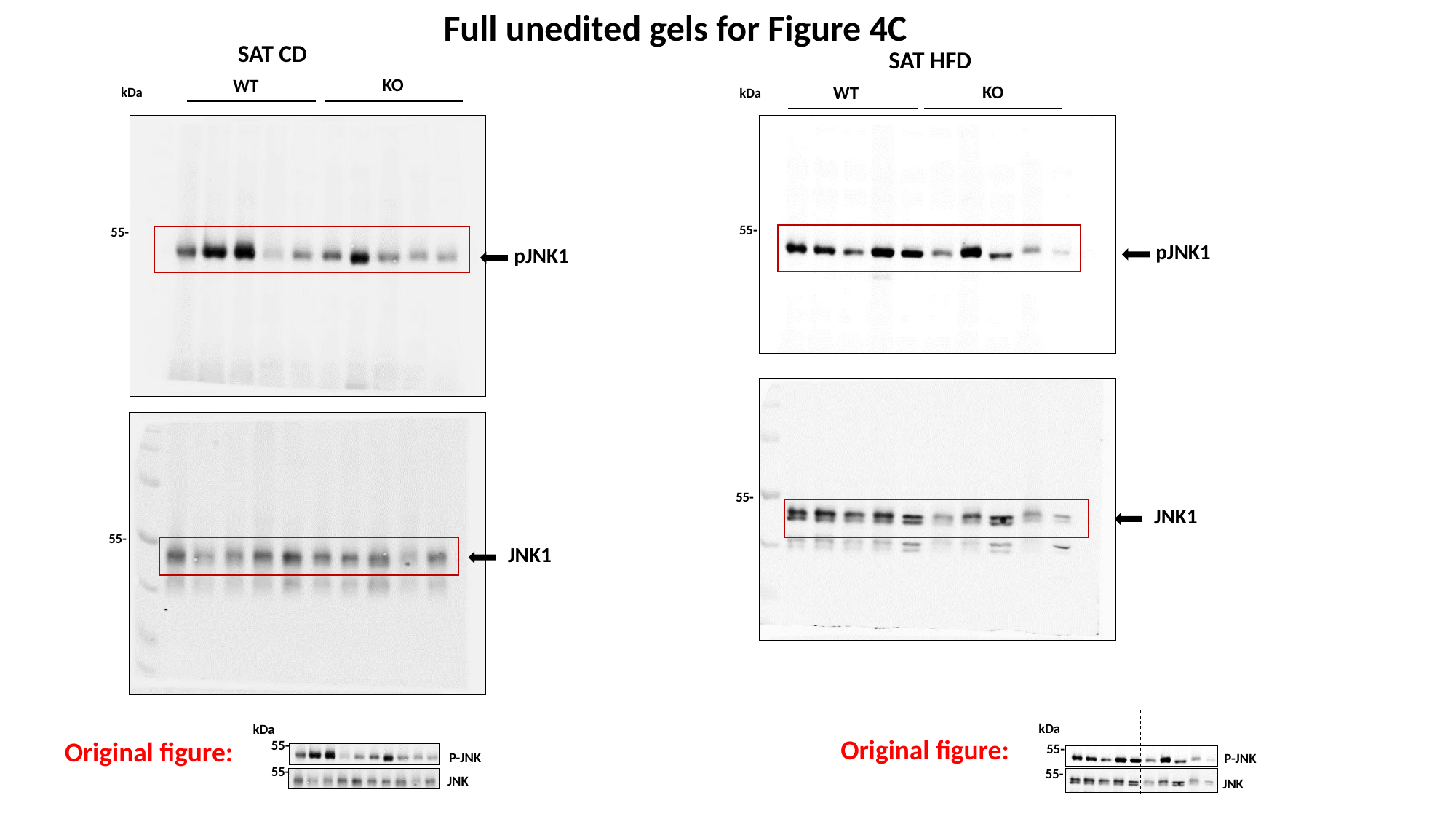

Full unedited gels for Figure 4C
SAT CD
SAT HFD
KO
WT
KO
WT
kDa
kDa
55-
55-
pJNK1
pJNK1
55-
JNK1
55-
JNK1
kDa
55-
P-JNK
55-
JNK
kDa
55-
P-JNK
55-
JNK
Original figure:
Original figure:

## Slide 11
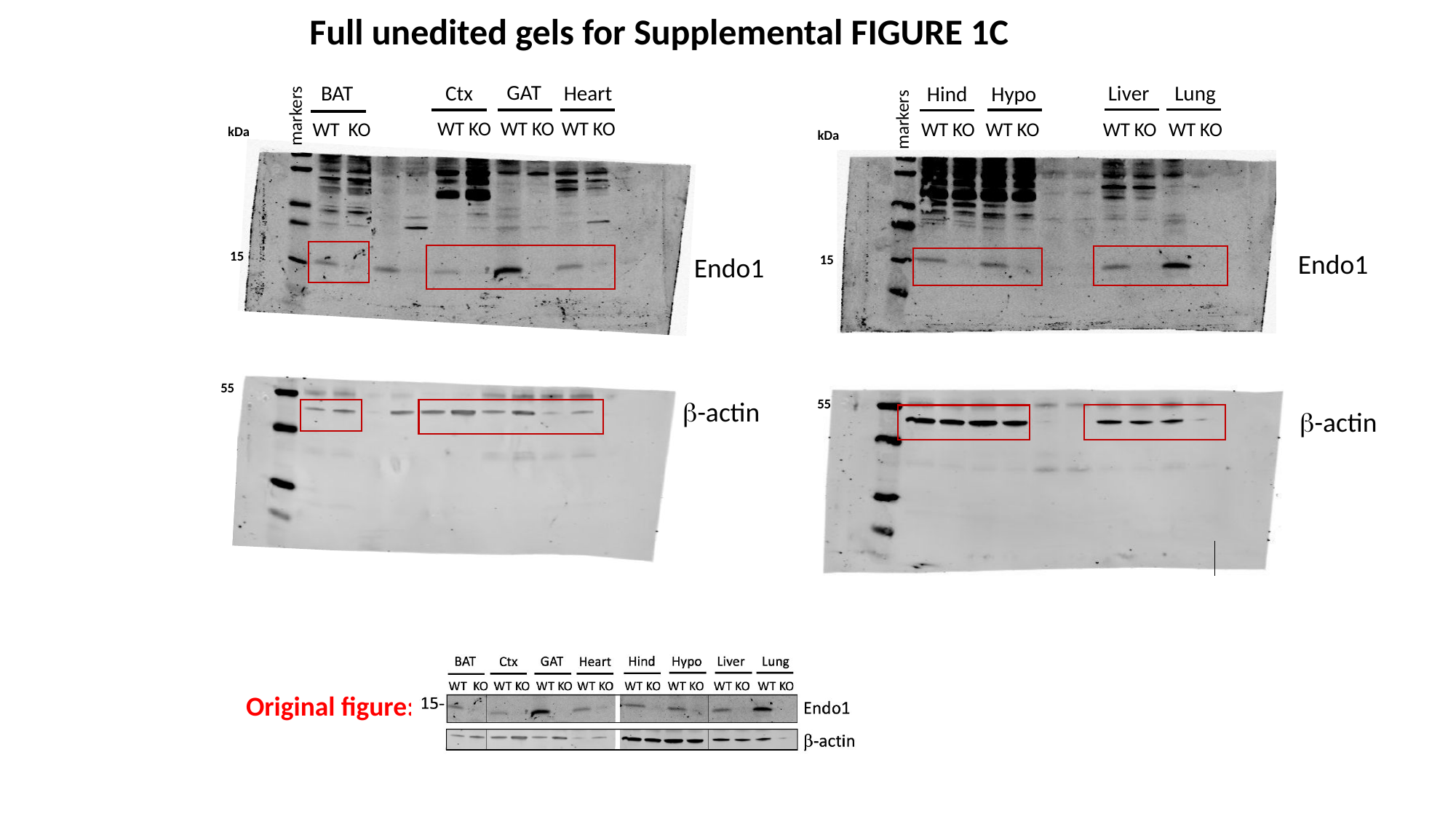

Full unedited gels for Supplemental FIGURE 1C
GAT
Ctx
Heart
Liver
Lung
BAT
Hind
Hypo
markers
markers
WT KO
WT KO
WT KO
WT KO
WT KO
WT KO
WT KO
WT KO
kDa
kDa
Endo1
15
Endo1
15
55
b-actin
55
b-actin
Original figure:

## Slide 12
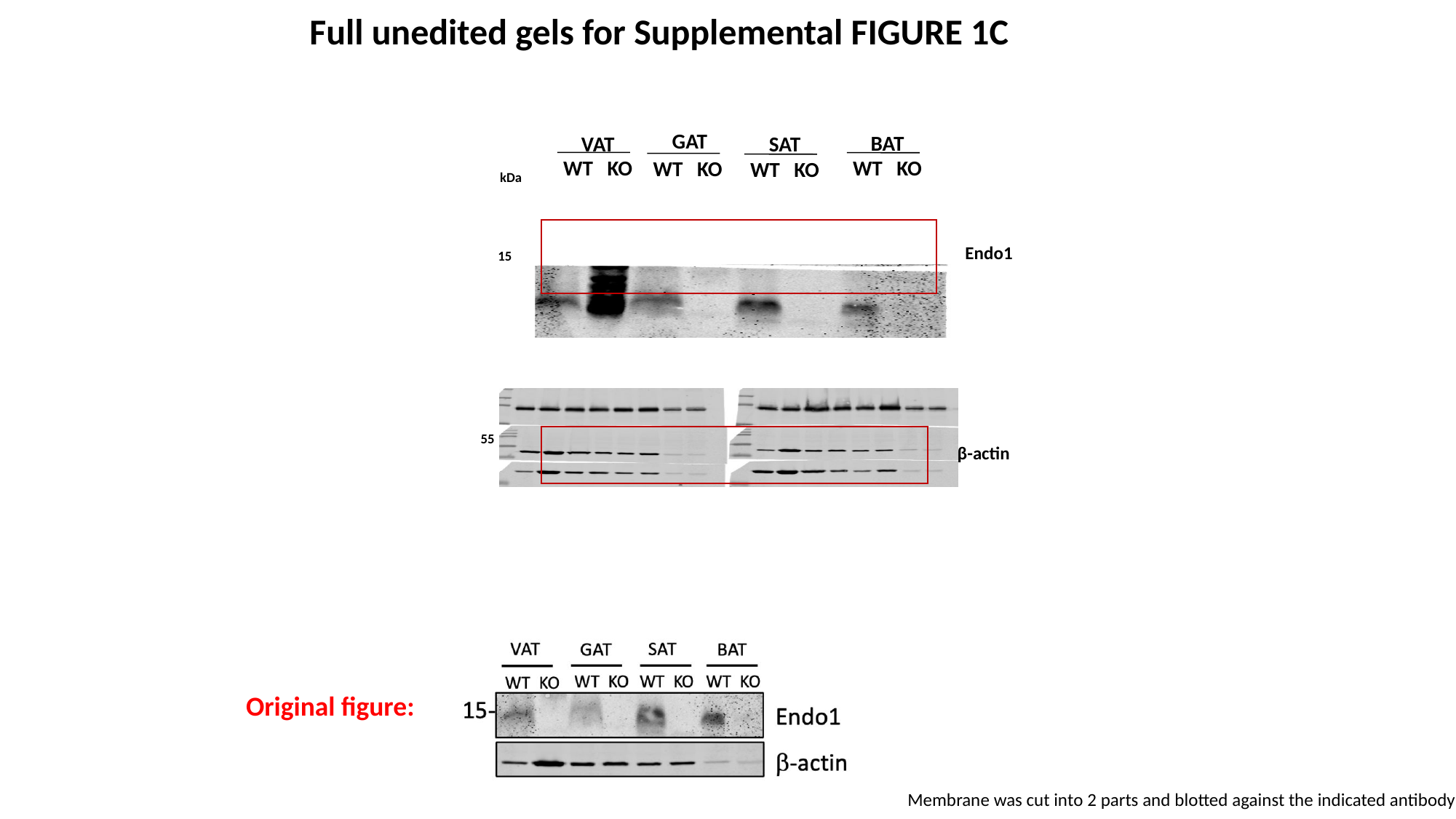

Full unedited gels for Supplemental FIGURE 1C
GAT
BAT
VAT
SAT
WT KO
WT KO
WT KO
WT KO
Endo1
β-actin
kDa
15
55
Original figure:
Membrane was cut into 2 parts and blotted against the indicated antibody

## Slide 13
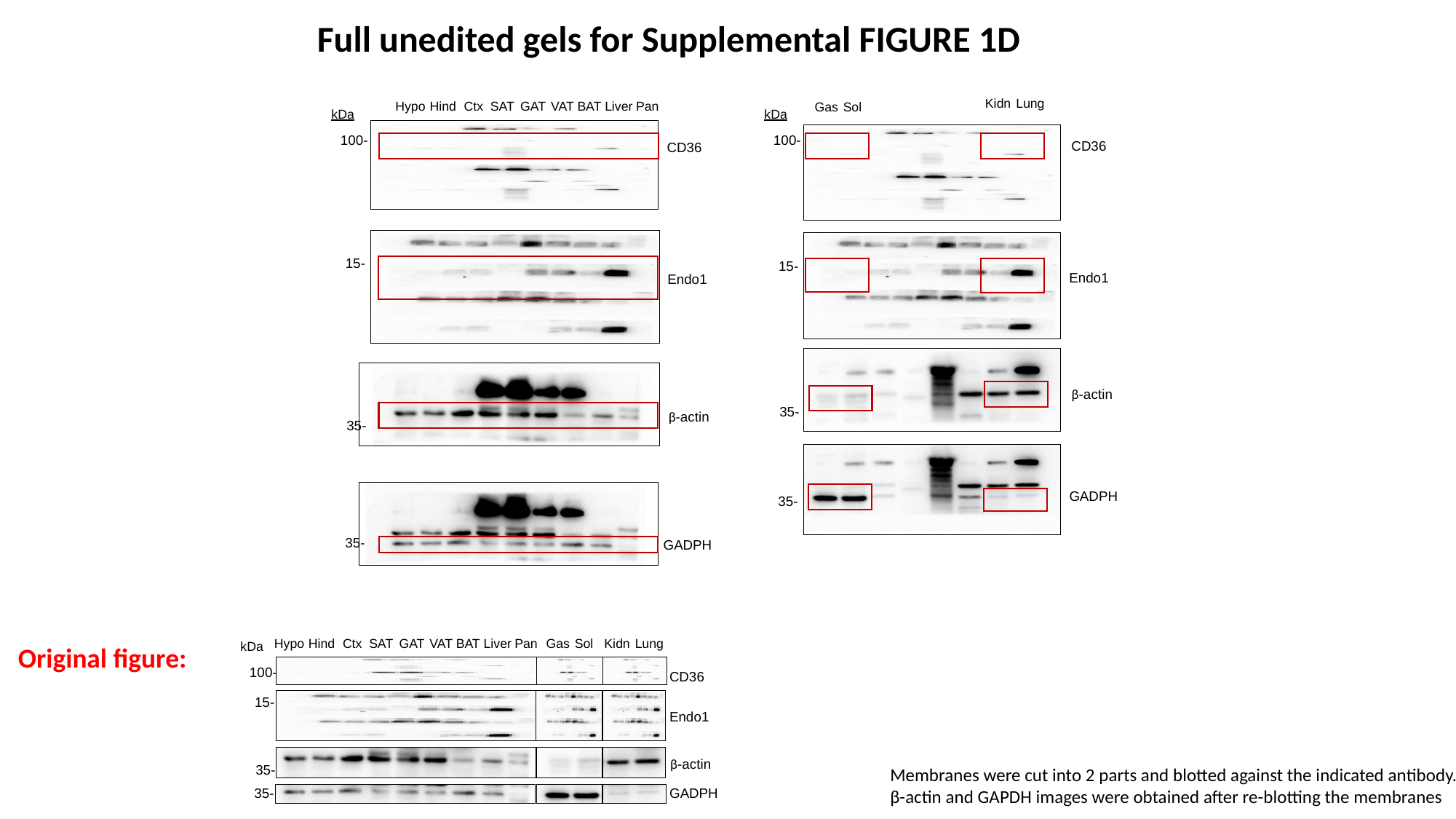

Full unedited gels for Supplemental FIGURE 1D
Kidn
Lung
Hypo
Hind
Ctx
SAT
GAT
VAT
BAT
Liver
Pan
Gas
Sol
kDa
kDa
100-
100-
CD36
CD36
15-
15-
Endo1
Endo1
β-actin
35-
β-actin
35-
GADPH
35-
35-
GADPH
Hypo
Hind
Ctx
SAT
GAT
VAT
BAT
Liver
Pan
Gas
Sol
Kidn
Lung
kDa
Original figure:
100-
CD36
15-
Endo1
β-actin
35-
Membranes were cut into 2 parts and blotted against the indicated antibody.
β-actin and GAPDH images were obtained after re-blotting the membranes
35-
GADPH

## Slide 14
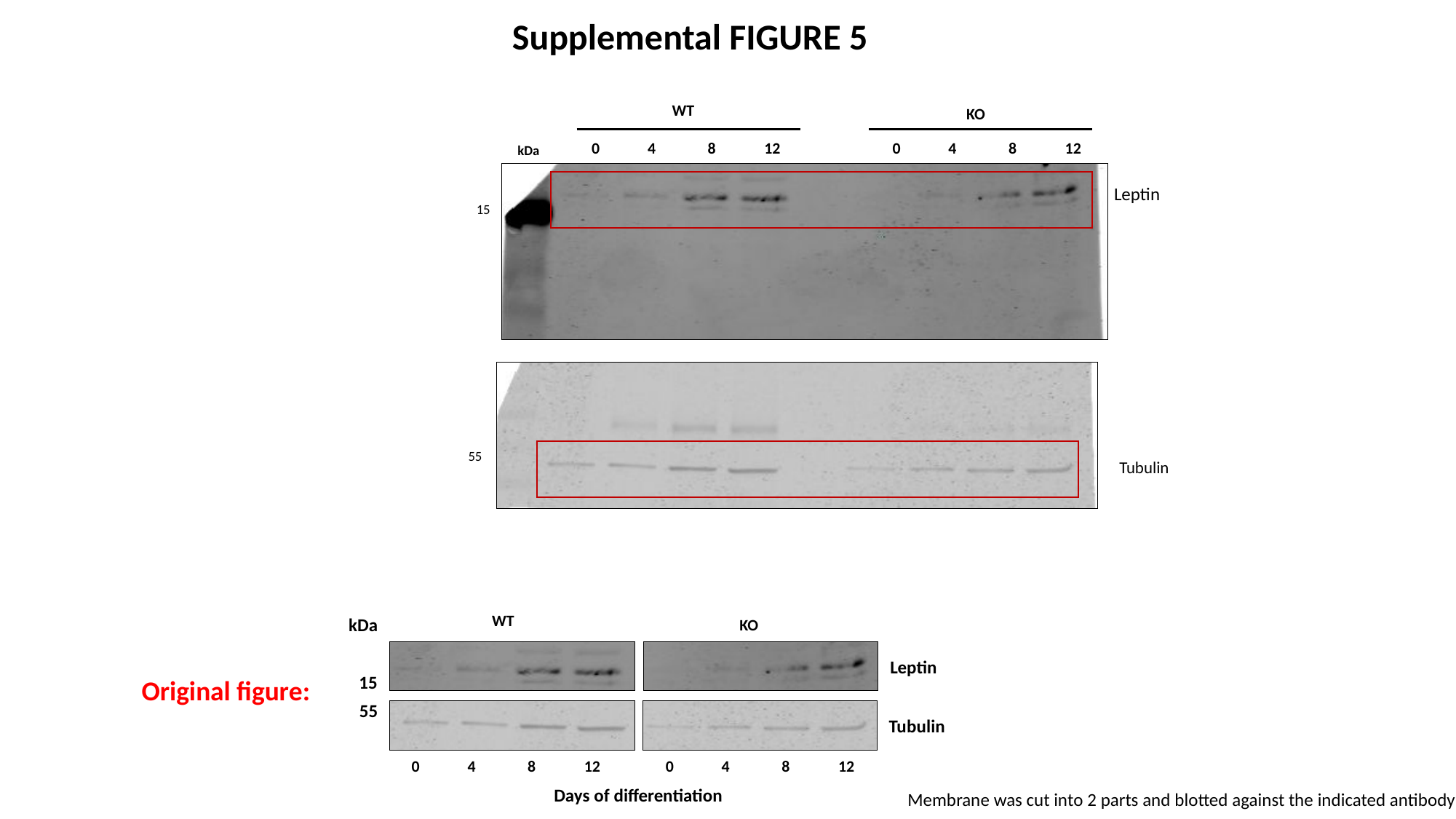

Supplemental FIGURE 5
WT
KO
0
4
8
12
0
4
8
12
kDa
Leptin
15
55
Tubulin
WT
kDa
KO
Leptin
15
55
Tubulin
0
4
8
12
0
4
8
12
Days of differentiation
Original figure:
Membrane was cut into 2 parts and blotted against the indicated antibody

## Slide 15
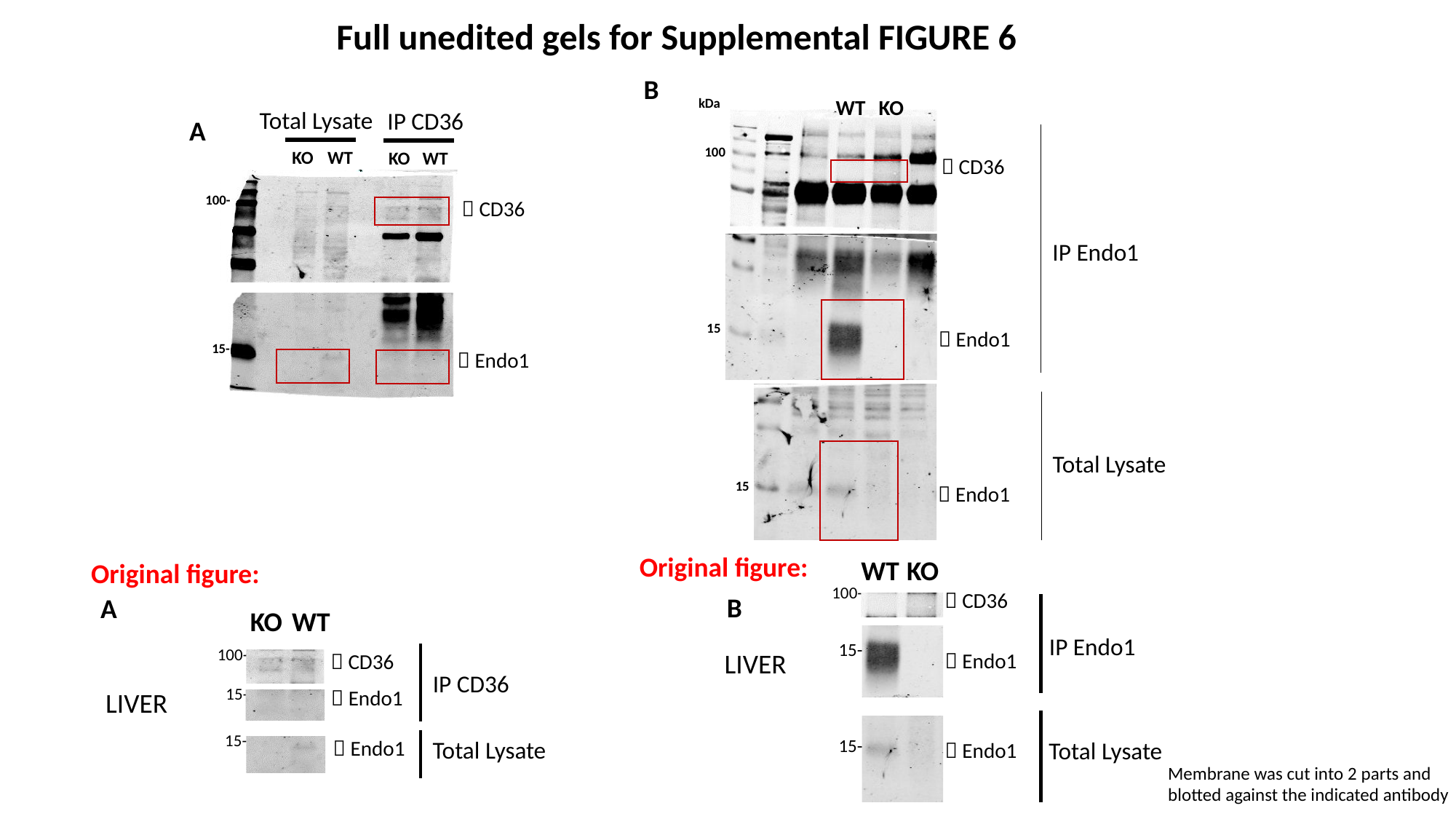

Full unedited gels for Supplemental FIGURE 6
B
KO
WT
kDa
Total Lysate
IP CD36
A
KO
WT
KO
WT
100-
 CD36
LIVER
15-
 Endo1
100
 CD36
IP Endo1
15
 Endo1
Total Lysate
15
 Endo1
Original figure:
WT
KO
100-
 CD36
IP Endo1
15-
LIVER
 Endo1
15-
Total Lysate
 Endo1
Original figure:
B
A
KO
WT
100-
 CD36
IP CD36
15-
 Endo1
LIVER
15-
 Endo1
Total Lysate
Membrane was cut into 2 parts and
blotted against the indicated antibody

## Slide 16
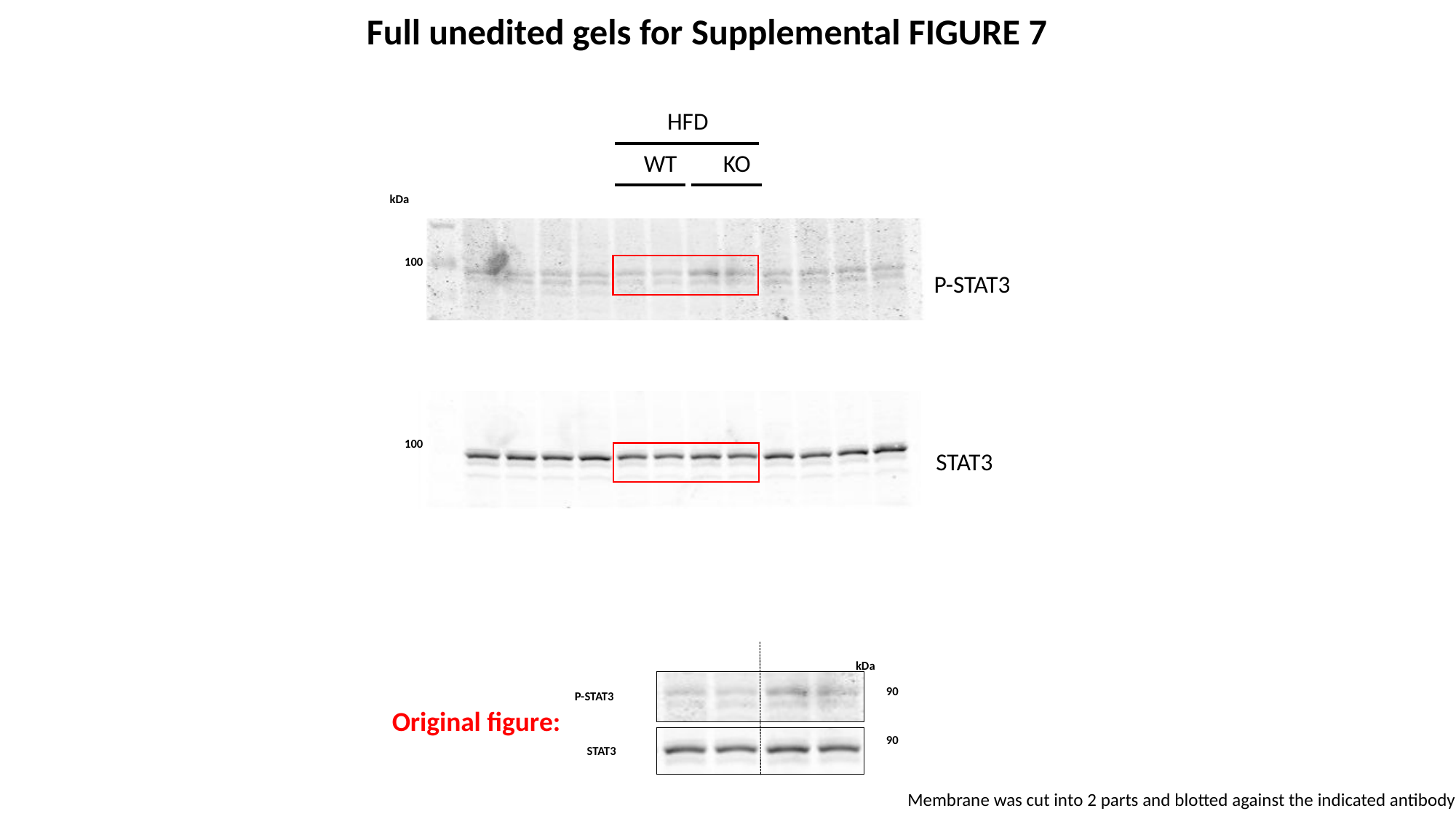

Full unedited gels for Supplemental FIGURE 7
HFD
WT
KO
kDa
100
P-STAT3
100
STAT3
kDa
90
P-STAT3
STAT3
90
Original figure:
Membrane was cut into 2 parts and blotted against the indicated antibody

## Slide 17
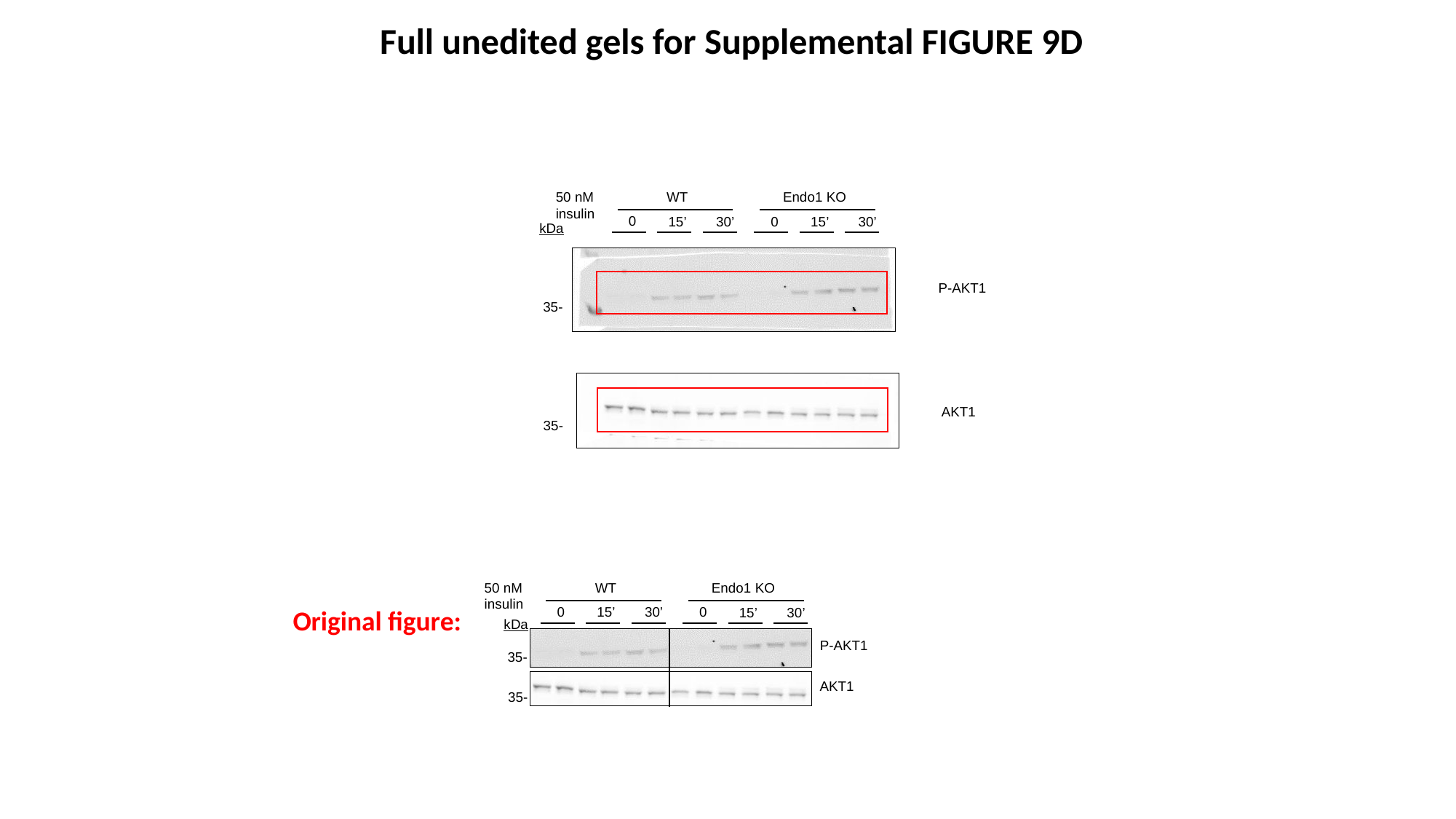

Full unedited gels for Supplemental FIGURE 9D
WT
Endo1 KO
50 nM
insulin
0
15’
30’
0
15’
30’
kDa
P-AKT1
35-
AKT1
35-
WT
Endo1 KO
50 nM
insulin
0
15’
30’
0
15’
30’
P-AKT1
AKT1
kDa
35-
35-
Original figure:

## Slide 18
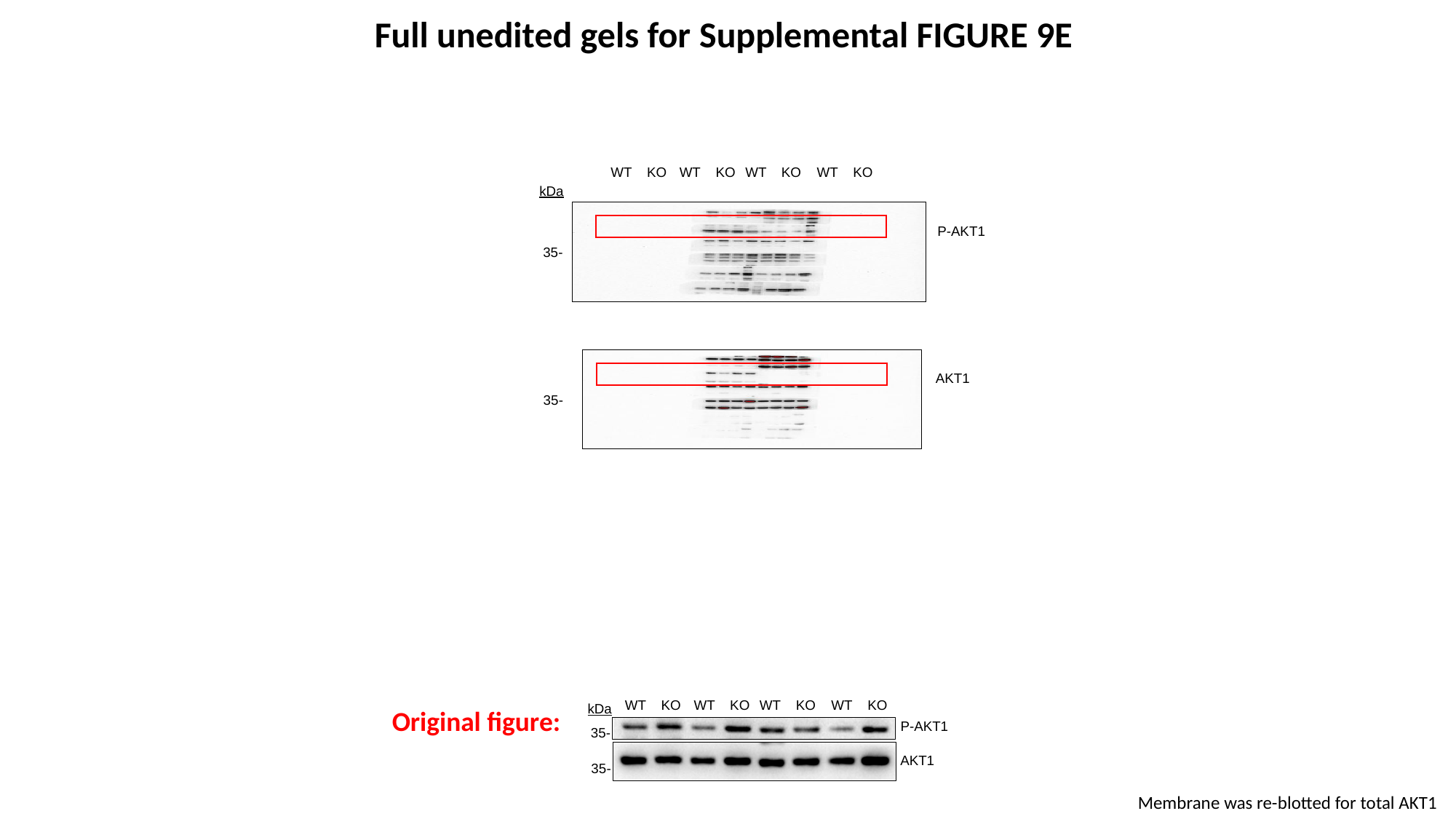

Full unedited gels for Supplemental FIGURE 9E
WT KO
WT KO
WT KO
WT KO
kDa
P-AKT1
35-
AKT1
35-
WT KO
WT KO
WT KO
WT KO
kDa
Original figure:
P-AKT1
35-
AKT1
35-
Membrane was re-blotted for total AKT1
